# PRC2.1- and PRC2.2-specific accessory proteins drive recruitment of different forms of canonical PRC1

**DOI:** 10.1016/j.molcel.2023.03.018

**Published:** 2023-05-04

**Authors:** Eleanor Glancy, Cheng Wang, Ellen Tuck, Evan Healy, Simona Amato, Hannah K. Neikes, Andrea Mariani, Marlena Mucha, Michiel Vermeulen, Diego Pasini, Adrian P. Bracken

**Affiliations:** 1 Smurfit Institute of Genetics, Trinity College Dublin, Dublin 2, Ireland; 2 Department of Experimental Oncology, IEO, European Institute of Oncology IRCCS, Via Adamello 16, 20139 Milan, Italy; 3 Department of Molecular Biology, Faculty of Science, Radboud Institute for Molecular Life Sciences, Oncode Institute, Radboud University Nijmegen, 6525 GA Nijmegen, the Netherlands; 4 Department of Health Sciences, University of Milan, Via A. di Rudini 8, 20142 Milan, Italy; 5 The Netherlands Cancer Institute, Amsterdam, The Netherlands

**Keywords:** Polycomb, PRC2.1, PRC2.2, H3K27me3, JARID2, Polycomb-like protein, PRC1, CBX2, CBX4, CBX7

## Abstract

Polycomb repressive complex 2 (PRC2) mediates H3K27me3 deposition, which is thought to recruit canonical PRC1 (cPRC1) via chromodomain-containing CBX proteins to promote stable repression of developmental genes. PRC2 forms two major subcomplexes, PRC2.1 and PRC2.2, but their specific roles remain unclear. Through genetic knockout (KO) and replacement of PRC2 subcomplex-specific subunits in naïve and primed pluripotent cells, we uncover distinct roles for PRC2.1 and PRC2.2 in mediating the recruitment of different forms of cPRC1. PRC2.1 catalyzes the majority of H3K27me3 at Polycomb target genes and is sufficient to promote recruitment of CBX2/4-cPRC1 but not CBX7-cPRC1. Conversely, while PRC2.2 is poor at catalyzing H3K27me3, we find that its accessory protein JARID2 is essential for recruitment of CBX7-cPRC1 and the consequent 3D chromatin interactions at Polycomb target genes. We therefore define distinct contributions of PRC2.1- and PRC2.2-specific accessory proteins to Polycomb-mediated repression and uncover a new mechanism for cPRC1 recruitment.

## Highlights

• PRC2.1/PRC2.2 are co-recruited to promote *de novo* Polycomb target gene repression
• PRC2.1 binds in sharp peaks and specializes in CBX2/4-cPRC1 recruitment
• PRC2.2 binds in broader H2AK119ub1-like profiles and has weak H3K27me3 acivity
• PRC2.2 component JARID2 specializes in CBX7-cPRC1 recruitment

## Graphical abstract

**Figure:**
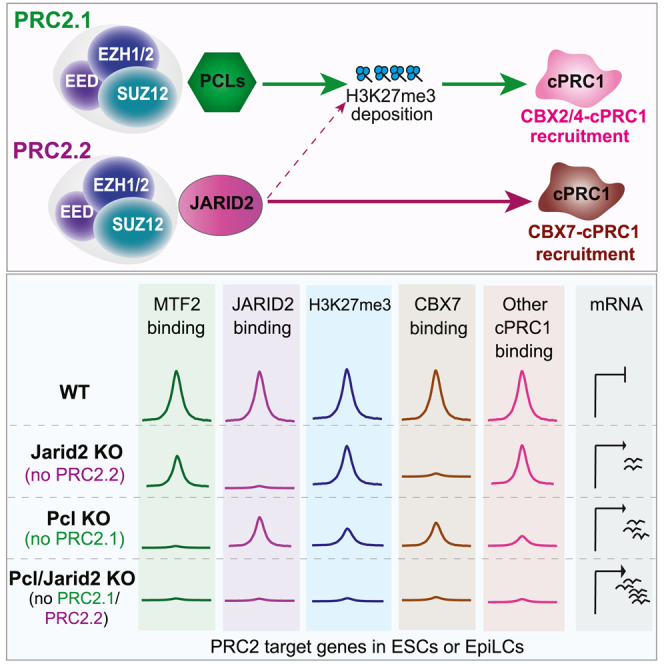

## Teaser

Glancy et al. reveal divergent molecular functions of PRC2 subcomplexes, PRC2.1 and PRC2.2, in mediating Polycomb target gene repression. PRC2.1 promotes the recruitment of CBX2/4-cPRC1 via H3K27me3 deposition, while PRC2.2 component JARID2 specializes in driving the recruitment of CBX7-cPRC1. These findings go a long way toward explaining why these two independent PRC2 subcomplexes have persisted throughout evolution.

## Introduction

Polycomb group proteins are chromatin-associated transcriptional repressors that are critical for maintaining cellular identity in higher eukaryotes.^1^^,^^2^^,^^3^^,^^4^ They contribute to establishing cellular identity by being recruited to and displaced from key lineage genes during cell-fate transitions.^5^^,^^6^^,^^7^^,^^8^^,^^9^ They function primarily as two multiprotein complexes, Polycomb repressive complex 1 (PRC1) and Polycomb repressive complex 2 (PRC2), both of which have histone-modifying activities.^10^ Many of the genes encoding Polycomb group proteins are essential for embryonic development, while mutations in PRC2 member genes are associated with human growth disorders^3^ and malignancies.^10^^,^^11^^,^^12^^,^^13^^,^^14^ Despite these central roles in development and disease, the distinct mechanisms by which PRCs function are still poorly understood.

It is thought that interplay between different forms of PRC1 and PRC2 mediates Polycomb silencing. PRC2 is built around core subunits EZH1/2, EED, and SUZ12, in association with the histone-binding RBBP4/7.^15^ The histone-methyl-transferases EZH1 and EZH2 are responsible for catalyzing mono-, di-, and tri-methylation of histone 3 at lysine 27 (H3K27me1/2/3) in higher eukaryotic cells.^15^^,^^16^^,^^17^ SUZ12 bridges the enzymatic core of PRC2 with several accessory subunits, which regulate its activity and recruitment to chromatin.^16^^,^^18^^,^^19^^,^^20^^,^^21^^,^^22^ Variant forms of PRC1 (vPRC1) catalyze mono-ubiquitination of histone H2A at lysine 119 (H2AK119ub1), which contributes to PRC2 binding and H3K27me3 deposition.^20^^,^^23^^,^^24^^,^^25^^,^^26^^,^^27^^,^^28^ The H3K27me3 modification is then thought to contribute to canonical PRC1 (cPRC1) recruitment via binding of chromodomain-containing CBX proteins.^29^^,^^30^^,^^31^ cPRC1 is then thought to contribute to stable gene repression via its 3D looping and chromatin compaction activities.^32^^,^^33^^,^^34^^,^^35^^,^^36^

PRC2 assembles into two mutually exclusive subcomplexes in mammals, PRC2.1 and PRC2.2.^37^^,^^38^^,^^39^^,^^40^ PRC2.1 contains one of three Polycomb-like proteins (PHF1, MTF2, or PHF19), together with either PALI1/2 or EPOP,^17^^,^^41^^,^^42^^,^^43^ while PRC2.2 contains JARID2 and AEBP2.^37^^,^^44^ PRC2.1 and PRC2.2 are targeted to largely the same loci in mouse embryonic stem cells (ESCs), where they combine to coordinate deposition of H3K27me3.^20^^,^^21^^,^^45^^,^^46^ The extended homologous (EH) domain of Polycomb-like proteins is thought to promote binding of PRC2.1 to CG-rich DNA within C-phosphate-G (CpG) islands (CGIs), but the sequence specificity of this interaction is disputed.^47^^,^^48^^,^^49^^,^^50^ Intriguingly, Polycomb-like proteins can also bind *in vitro* to H3K36me2/3 and more weakly to H3K27me3 via their N-terminal Tudor domain.^51^^,^^52^^,^^53^^,^^54^ However, the potential functional relevance of these interactions remains unknown.^16^ In contrast to PRC2.1, PRC2.2 members JARID2 and AEBP2 bind to H2AK119ub1, which enhances PRC2.2 histone-methyltransferase activity *in vitro*.^26^ A ubiquitin-interacting motif (UIM) at the N terminus of JARID2 and an H2AK119ub1 binding pocket in AEBP2 are required for this interaction.^24^^,^^55^ Supporting the link between PRC2.2 and H2AK119ub1, mouse ESCs with depleted H2AK119ub1 display a stronger reduction of PRC2.2 binding to chromatin, compared with PRC2.1.^20^^,^^23^^,^^25^^,^^28^^,^^56^ However, despite emerging evidence of potential divergent roles during differentiation,^57^^,^^58^ it is unclear why two PRC2 subcomplexes have persisted throughout evolution.

Here, we applied genetic and quantitative genomic approaches to study the roles of PRC2.1 and PRC2.2, using a model of naive to primed pluripotency.^59^^,^^60^ This revealed that PRC2.1, PRC2.2, and cPRC1 are universally co-displaced from, and co-recruited to, Polycomb target genes during this cell-fate transition. Strikingly, we discovered distinct functions for PRC2.1- and PRC2.2-specific subunits in ESCs and during differentiation. While PRC2.1 is the dominant subcomplex for promoting the deposition of H3K27me3 at target genes in ESCs and epiblast-like cells (EpiLCs), we found that it is not sufficient to promote CBX7-cPRC1 recruitment and that it instead promotes the recruitment of CBX2-cPRC1 and CBX4-cPRC1. By contrast, although PRC2.2 only weakly contributes to H3K27me3 deposition, we show that JARID2 functions to drive CBX7-cPRC1 recruitment to Polycomb target genes. Our results assign independent functions to PRC2.1- and PRC2.2-specific accessory proteins and challenge the prevailing model of cPRC1 recruitment.

## Results

### Co-recruitment and co-displacement of PRC2.1 and PRC2.2 during transition from naive to primed pluripotency

**Figure 1:**
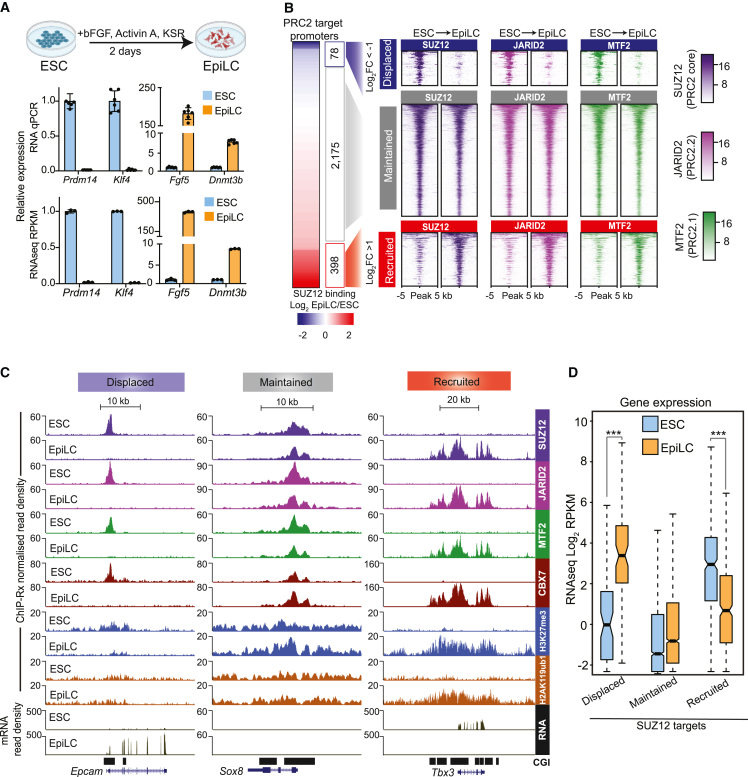
Co-recruitment and co-displacement of PRC2.1 and PRC2.2 during ESC to EpiLC differentiation (A) Top: schematic of differentiation model. Bottom: bar plots showing the expression of ESC marker genes *Prdm14* and *Klf4*, and EpiLC marker genes *Fgf5* and *Dnmt3b* by qPCR (n = 2) and RNA-seq (n = 3). Error bars represent SD. (B) Left: heatmap representing fold change in SUZ12 binding at PRC2 target promoters in ESC versus EpiLC cells. Indicated are three categories of PRC2 targets—displaced SUZ12 (log_2_FC < −1 and p value < 0.05; n = 78), maintained SUZ12 (n = 2,175), and recruited SUZ12 in EpiLC cells (log_2_FC > 1 and p value < 0.05; n = 398). Right: tornado plots showing enrichments of indicated antibodies at displaced, maintained, and recruited promoters in ESCs and EpiLCs. (C) Genome browser tracks showing ChIP-Rx for the indicated antibodies and RNA-seq profiles in ESC and EpiLC cells at *Epcam* (displaced), *Sox8* (maintained), and *Tbx3* (recruited). (D) Boxplots presenting mRNA abundance of displaced, maintained, and recruited PRC2 target genes. ^∗∗∗^p value < 0.001. See also Figure S1 and Table S1.

To explore the occupancy and dynamics of PRC2.1 and PRC2.2 subcomplexes during differentiation, we induced ESCs to undergo directed differentiation to post-implantation pre-gastrulation EpiLCs.^59^ We confirmed this through downregulation of genes associated with naive pluripotency, including *Prdm14* and *Klf4*, and upregulation of genes associated with primed pluripotency, including *Fgf5* and *Dnmt3b* (Figure 1A). A key strength of this system for studying PRC2.1 and PRC2.2 is that the levels of MTF2 and JARID2 are stable during the 2-day directed differentiation (Figures S1A and S1B).

We performed quantitative chromatin immunoprecipitation sequencing (ChIP-seq) with exogenous reference genome spike-in (ChIP-Rx) of PRC2.1- and PRC2.2-specific subunits—MTF2 and JARID2, respectively—as well as the core PRC2 member SUZ12 (Figure 1B). We subdivided PRC2-bound target promoters based on fold changes in SUZ12 abundance between ESCs and EpiLCs, thereby generating three categories: those with displaced SUZ12 (n = 78), the majority that maintained SUZ12 (n = 2,175), and those that recruited SUZ12 (n = 398) (Figure 1B; Table S1). This revealed a remarkable co-recruitment and co-displacement of PRC2.1 and PRC2.2 during ESC-EpiLC differentiation (Figures 1B and S1C). Genome browser tracks of representative genes from each of the three categories are highlighted (Figure 1C). Further supporting the co-dynamics of PRC2.1 and PRC2.2, we did not find any evidence of specific recruitment of either subcomplex to unique sites in EpiLCs (Figure S1C). Notably, we also observed an accumulation of H3K27me3, vPRC1-mediated H2AK119ub1, and cPRC1 member CBX7 on PRC2-recruited genes (Figures 1C and S1D).

Next, we examined the mRNA levels of the three groups of Polycomb target genes during ESC-EpiLC differentiation. The expression of genes with PRC2.1/PRC2.2 displacement was increased, and this correlated with an accumulation of H3K27ac, whereas the expression of genes with co-recruitment of PRC2.1/PRC2.2 was repressed and correlated with a depletion of H3K27ac (Figures 1D and S1D). The maintained group of target genes, which had no change in PRC2.1 or PRC2.2 binding, remained in their repressed states both before and after differentiation (Figure 1D). Taken together, these data suggest that during the transition from naive to primed pluripotency, the co-displacement and co-recruitment of PRC2.1 and PRC2.2 are directly associated with transcriptional upregulation and downregulation of target genes, respectively.

### PRC2.1 drives H3K27me3 deposition while PRC2.2 component JARID2 drives CBX7-cPRC1 recruitment at *de novo* target genes

**Figure 2:**
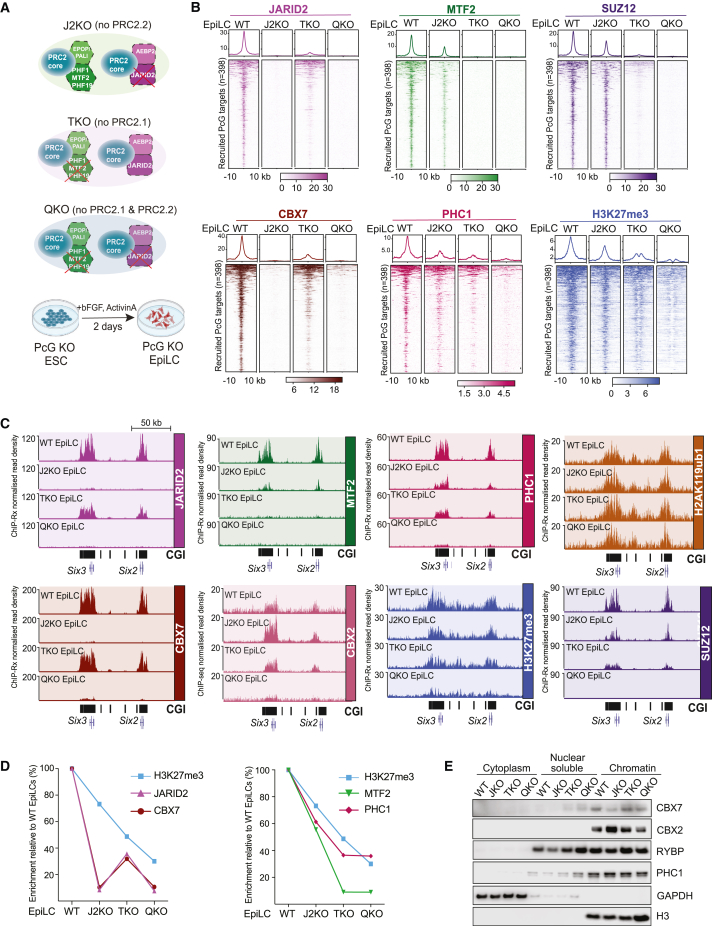
PRC2.1 drives H3K27me3 deposition while PRC2.2 drives CBX7-cPRC1 recruitment to Polycomb target genes (A) Schematic of ESC lines used. (B) Tornado and average plots showing ChIP-Rx enrichments for the indicated antibodies at recruited Polycomb target genes (n = 398 sites) in WT and mutant EpiLCs. (C) Genome browser tracks showing ChIP-Rx profiles of the indicated antibodies in WT and mutant EpiLCs at the maintained *Six3* and *Six2* gene loci. (D) Line plots representing ChIP-Rx enrichment of indicated antibodies in WT and mutant EpiLCs, relative to their respective levels in WT EpiLCs. (E) Western blot of the indicated antibodies on cytoplasm, nucleosol, or chromatin fractions of the indicated cell lines. See also Figure S2.

Next, we wanted to explore the relative contributions of PRC2.1 and PRC2.2 during this cell-state transition. To address this, we used a set of ESC lines we developed previously,^20^ including “WT” (wild-type), “TKO” (lacking the three paralogous Polycomb-like proteins; PHF1, MTF2, and PHF19), “J2KO” (lacking JARID2), and “QKO” (lacking the three Polycomb-like proteins and JARID2), and induced them to differentiate to EpiLCs (Figure 2A). We again used ChIP-Rx to analyze the enrichment of core PRC2 subunit SUZ12, PRC2.2-specific JARID2, PRC2.1-specific MTF2, cPRC1 subunits CBX7 and PHC1, and PRC2-mediated H3K27me3 at the Polycomb-recruited sites (Figures 2B and S2A) and at sites that maintained Polycomb occupancy during the ESC-EpiLC differentiation (Figures 2C and S2B). The loss of PRC2.1 (TKO) had a stronger negative impact on H3K27me3 accumulation, compared with the loss of PRC2.2 function (J2KO), while the combined loss of both (QKO) reduced it to minimal levels (Figures 2B, 2C, S2A, and S2B). Notably, loss of the enzymatically dominant PRC2.1 in TKO and QKO cells caused H3K27me3 to focus around two peaks (Figure 2B). Intriguingly, a strong co-dependence between the two subcomplexes is evident from the fact that loss of JARID2 leads to reduced MTF2 recruitment and vice versa (Figures 2B, 2C, S2A, and S2B). The combined loss of JARID2 and Polycomb-like proteins was required for complete ablation of PRC2 recruitment and almost complete ablation of H3K27me3 enrichment (Figures 2B, 2C, S2A, and S2B), supporting previous results in ESCs.^20^^,^^21^^,^^45^

Remarkably, loss of PRC2.2-specific subunit JARID2 reduced CBX7-cPRC1 binding to background levels, whereas the loss of PRC2.1-specific MTF2 caused a much more moderate reduction in CBX7-cPRC1 binding at both increased and maintained target genes (Figures 2B, 2C, and S2A). This was particularly surprising since loss of JARID2 had a minimal impact on H3K27me3 accumulation (Figures 2B, 2C, S2A, and S2B). We also performed ChIP-Rx of CBX7 in EpiLCs generated from an independent *Jarid2*-null line and confirmed our observations (Figure S2C). We further confirmed the dependency of CBX7 on JARID2 in *Jarid2*-null ESCs (Figure S2D) and with an alternative CBX7 antibody (Figure S2E). Furthermore, we observed that disruption of CBX7-cPRC1 through *Pcgf2/4*-null EpiLCs did not affect JARID2 binding (Figure S2F), confirming that JARID2 acts upstream of CBX7-cPRC1. Since loss of JARID2 did not affect the stability of *Cbx7* mRNA or protein levels (Figures S2G and S2H), these data imply that JARID2 is necessary for enrichment of CBX7 at Polycomb target genes.

Since PHC1 is the most abundant PHC subunit in cPRC1 in ESCs,^4^ we next performed ChIP-Rx of PHC1 in mutant and matched WT ESCs and EpiLCs (Figures 2B, 2C, S2A, and S2B). This revealed that in contrast to CBX7, which tracks with JARID2, PHC1 tracked more closely with MTF2 and H3K27me3 (Figures 2B–2D). This suggests that most cPRC1 complexes are predominantly dependent on the levels of H3K27me3 for their association with target genes. This prompted us to investigate the effects of loss of PRC2.1 and PRC2.2 on the recruitment of the other CBX proteins. Since *Cbx4* is not expressed in EpiLCs (Figure S1B), we performed ChIP-seq of CBX2 in mutant and matched WT EpiLCs (Figures 2C and S2B). This strikingly revealed that CBX2 had increased binding on Polycomb target genes in *Jarid2*-null cells. Furthermore, in contrast to CBX7, the total amount of CBX2 binding to chromatin is increased in *Jarid2*-null ESCs (Figure 2E). However, in QKO ESCs that lack *Jarid2* and the three Polycomb-like proteins, CBX2 binding was mostly lost from Polycomb target genes, likely as a consequence of the reduced H3K27me3. Finally, we found that vPRC1-mediated H2AK119ub1 accumulation was largely unchanged in the absence of PRC2 subcomplexes (Figures 2C and S2B), consistent with it acting upstream of PRC2 recruitment.^20^^,^^23^^,^^28^

Taken together, these data demonstrate that the PRC2.2 subunit JARID2 drives CBX7-cPRC1 recruitment to Polycomb target genes, whereas other cPRC1 formations are more reliant on H3K27me3, which is predominantly deposited by PRC2.1.

### JARID2 drives CBX7-cPRC1 recruitment while MTF2 drives CBX4-cPRC1 recruitment in ESCs

**Figure 3:**
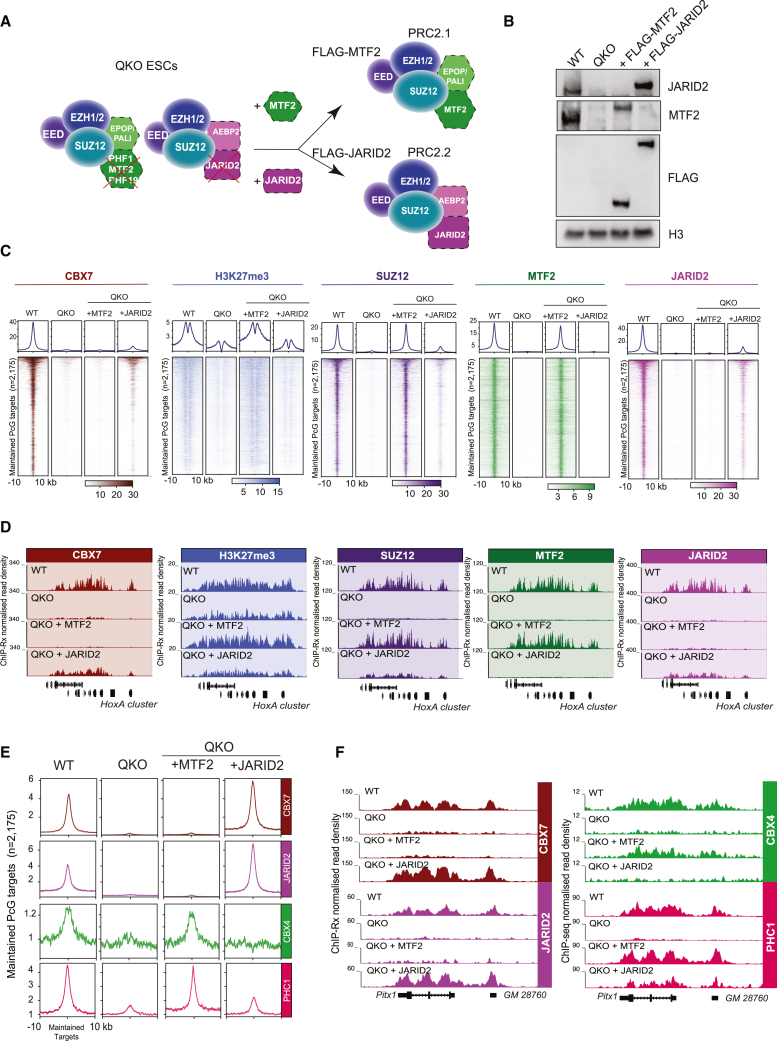
JARID2 promotes CBX7-cPRC1 while MTF2 promotes CBX4-cPRC1 recruitment to Polycomb target genes in ESCs (A) Schematic of PRC2.1 or PRC2.2 rescue strategy in QKO ESCs. (B) Western blot analyses of the indicated antibodies on total protein extracts from QKO ESC rescue lines, described in (A). (C) Average and tornado plots showing ChIP-Rx enrichments of indicated antibodies at maintained Polycomb targets (n = 2,175) in the relevant cell lines. (D) Genome browser tracks showing ChIP-Rx enrichments of indicated antibodies in the relevant cells at the extended *HoxA* locus. (E) Average plot showing ChIP-Rx and ChIP-seq enrichments of indicated antibodies at maintained Polycomb target genes (n = 2,175) in the relevant cell lines. (F) Genome browser tracks showing ChIP-Rx and ChIP-seq enrichments of indicated antibodies in the relevant cell lines at the *Pitx1* locus. See also Figure S3.

To further explore the respective functions of PRC2.1 and PRC2.2 in H3K27me3 deposition and cPRC1 recruitment, we established an exogenous *de novo* recruitment assay in mouse ESCs (Figure 3A). We ectopically expressed either FLAG-tagged MTF2 or FLAG-tagged JARID2 in QKO ESCs (Figure 3B). Importantly, expression of FLAG-MTF2 or FLAG-JARID2 was sufficient to re-establish PRC2.1 and PRC2.2 formation, respectively (Figure S3A), and Polycomb target gene binding (Figure S3B). Our previous work established that the majority of Polycomb target genes in ESCs are co-occupied by PRC2.1 and PRC2.2, while a subgroup of 187 gene promoters was bound by PRC2.1 only.^20^ Confirming the specificity of our assay, we found that the exogenous expression of FLAG-MTF2 promoted PRC2.1 binding at both “shared” and “PRC2.1 only” sites, whereas expression of FLAG-JARID2 promoted binding solely at the shared sites (Figure S3C). Consistent with its inability to bind to PRC2.1 only sites, FLAG-JARID2 was only capable of promoting CBX7 binding on PRC2.1/PRC2.2 shared sites (Figure S3C).

Strikingly, although exogenous FLAG-MTF2 promoted H3K27me3 deposition, it did not lead to CBX7 recruitment, whereas FLAG-JARID2 promoted CBX7 recruitment without promoting increases in H3K27me3 levels (Figures 3C and 3D). These results suggest that CBX7-cPRC1 is recruited, at least partially, independently of high levels of H3K27me3. Next, to directly compare the respective abilities of MTF2 and JARID2 to recruit CBX4-cPRC1 and CBX7-cPRC1 to target genes in ESCs, we repeated and extended the ectopic rescue assays (Figures 3E and 3F). In this experiment, we achieved higher levels of FLAG-JARID2, and this correlated with increased recruitment of CBX7 (Figures 3E and 3F). Strikingly, although FLAG-MTF2 was again not capable of promoting CBX7 recruitment, it was capable of recruiting CBX4 and PHC1 (Figures 3E and 3F). We believe this is due to a greater dependence of CBX4-cPRC1 on H3K27me3, which is largely mediated by MTF2-PRC2.1 (Figure 3C). It is also clear that PHC1 increases slightly upon FLAG-JARID2 expression, and this is likely representative of CBX7-PHC1-cPRC1 complexes (Figures 3E, 3F, and S3D).

We next speculated that PRC2-mediated methylation of residue K116 in JARID2 could serve as a binding substrate for the CBX7 chromodomain.^61^^,^^62^ To test this, we ectopically expressed either WT JARID2 or a JARID2-K116R mutant in QKO ESCs (Figure S3E). This revealed that the K116R mutation had no effect on the ability of JARID2 to promote CBX7 recruitment to target genes, and it suggests further that CBX7 localization is not largely affected by partially impaired PRC2.2 enzymatic activity. We next used *Aebp2* knockout (KO) ESCs^44^ and found that loss of AEBP2 has no consequence on the recruitment of CBX7 to target genes (Figures S3F and S3G). Furthermore, to rule out the possibility that JARID2 and CBX7 directly interact, we performed endogenous immunoprecipitation (IP) mass spectrometry of CBX7 in WT ESCs. This revealed that while we could detect the expected cPRC1 components, including PHC1/2/3 and PCGF2/4, we did not detect any PRC2 components above immunoglobulin G (IgG) negative control (Figure S3H).

Taken together, we have uncovered divergent functions for accessory proteins of PRC2.1 and PRC2.2 in the recruitment of different versions of cPRC1.

### CBX7-cPRC1 requires both JARID2 and H3K27me3 to bind to Polycomb target genes

**Figure 4:**
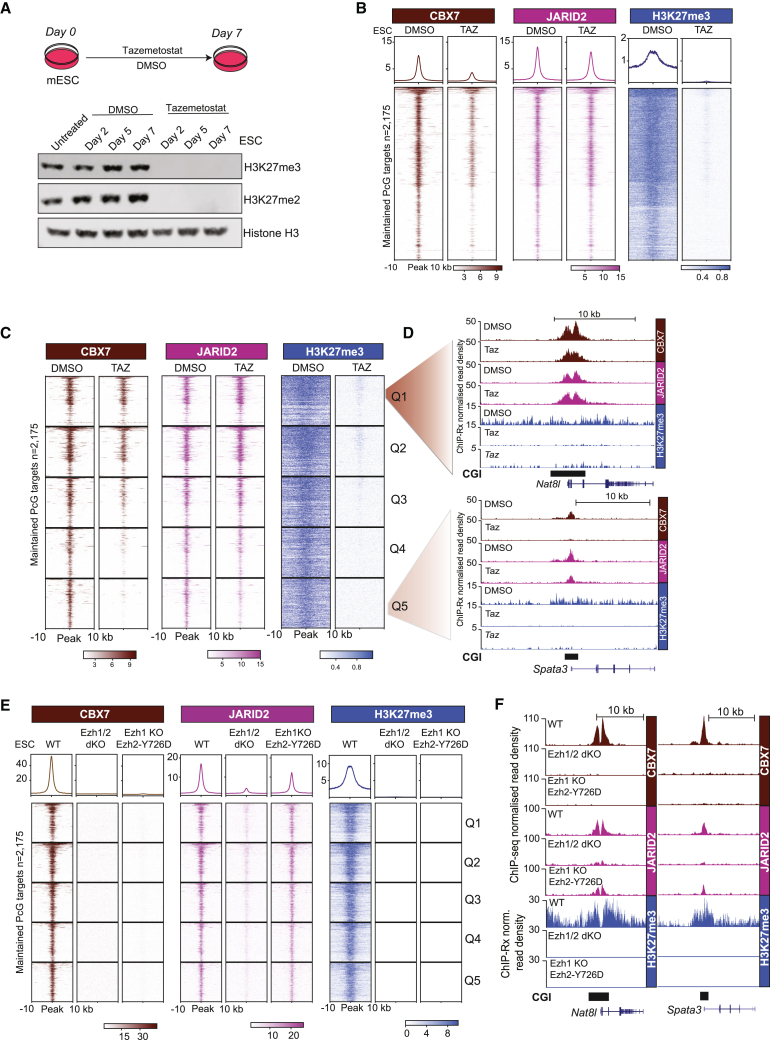
CBX7-cPRC1 requires JARID2 and low levels of H3K27me3 to bind to Polycomb target genes (A) Top: schematic of experimental design. Bottom: western blot analyses for the indicated antibodies on total protein extracts. (B) Average and tornado plots showing ChIP-Rx enrichments of indicated antibodies at maintained Polycomb targets (n = 2,175) in tazemetostat-treated or DMSO control ESCs. (C) Tornado plots showing ChIP-Rx enrichments of indicated antibodies in tazemetostat-treated or DMSO control ESCs at maintained Polycomb targets (n = 2,175), grouped into quintiles based on CBX7 abundance difference between DMSO and tazemetostat-treated ESCs. (D) Genome browser tracks of representative genes from quintile 1 (*Nat8I*) and quintile 5 (*Spata3*), showing ChIP-Rx of indicated antibodies in tazemetostat-treated or DMSO control ESCs. (E) Average and tornado plots showing ChIP-seq and ChIP-Rx enrichments of indicated antibodies in WT, *Ezh1*/*2*-dKO, and *Ezh1* KO/*EZH2-Y726D* at maintained Polycomb targets (n = 2,175) grouped into quintiles, as described in (C). (F) Genome browser tracks of indicated antibodies in *Ezh1*/*2*-dKO and *Ezh1* KO/*EZH2-Y726D* at representative genes from Q1 (*Nat8I*) and Q5 (*Spata3*). See also Figure S4.

To determine if H3K27me3 is required for CBX7-cPRC1 binding to Polycomb target genes, we treated WT ESCs with an inhibitor of PRC2 histone-methyltransferase activity (tazemetostat) for 7 days (Figure 4A). To test how this impacted the genome-wide occupancy and activities of PRC1 and PRC2, we performed genome-wide ChIP-Rx of H3K27me3, JARID2 and CBX7 (Figures 4B–4D) and SUZ12, MTF2 and H2AK119ub1 (Figures S4A–S4C). This revealed that even though CBX7 is overall reduced, it is not displaced from all target genes (Figure 4B), while tazemetostat effects on JARID2 binding were mild (Figure 4B). Interestingly, SUZ12, MTF2, and H2AK119ub1 increased on target genes (Figures S4A and S4B). We next divided Polycomb target genes into five quintiles, based on the CBX7 enrichment difference between DMSO and tazemetostat-treated cells, and plotted ChIP-Rx of CBX7, JARID2, and H3K27me3 (Figures 4C, 4D, and S4C). This revealed that CBX7 was largely retained on sites in quintile 1, while completely depleted from sites in quintile 5, and that its relative reduction on target genes correlated with reductions of JARID2 (Figures 4C, 4D, and S4C). However, an inherent limitation of this pharmacological approach, using an S-Adenosyl-L-methionine-competitive inhibitor, is that H3K27me3 is not 100% removed on all Polycomb target genes (Figure 4C).

This prompted us to take a genetic approach to evaluating if the complete loss of H3K27me3 would affect CBX7 binding to target genes. We availed of an ESC line CRISPR engineered to express a catalytically dead *Ezh2* (*Ezh2-Y726D*) in an *Ezh1* KO background.^63^ We performed ChIP-Rx of H3K27me3 and ChIP-seq of SUZ12, JARID2, and CBX7 (Figures 4E, 4F, S4D, and S4E). This confirmed that we could induce a complete loss of H3K27me3 in matched *Ezh1/2* KO and *Ezh1* KO/*Ezh2-Y726D* mutant ESCs lines (Figures 4E and 4F). Importantly, while SUZ12 and JARID2 were present on Polycomb target genes in this latter context, confirming the formation of a stable PRC2, no CBX7 was bound to Polycomb target genes.

Taken together, we propose that while modifying the levels of JARID2 on Polycomb target genes controls CBX7-cPRC1 recruitment, the presence of H3K27me3 is also necessary.

### Contrasting PRC2.1 and PRC2.2 binding profiles are consistent with independent recruitment mechanisms

**Figure 5:**
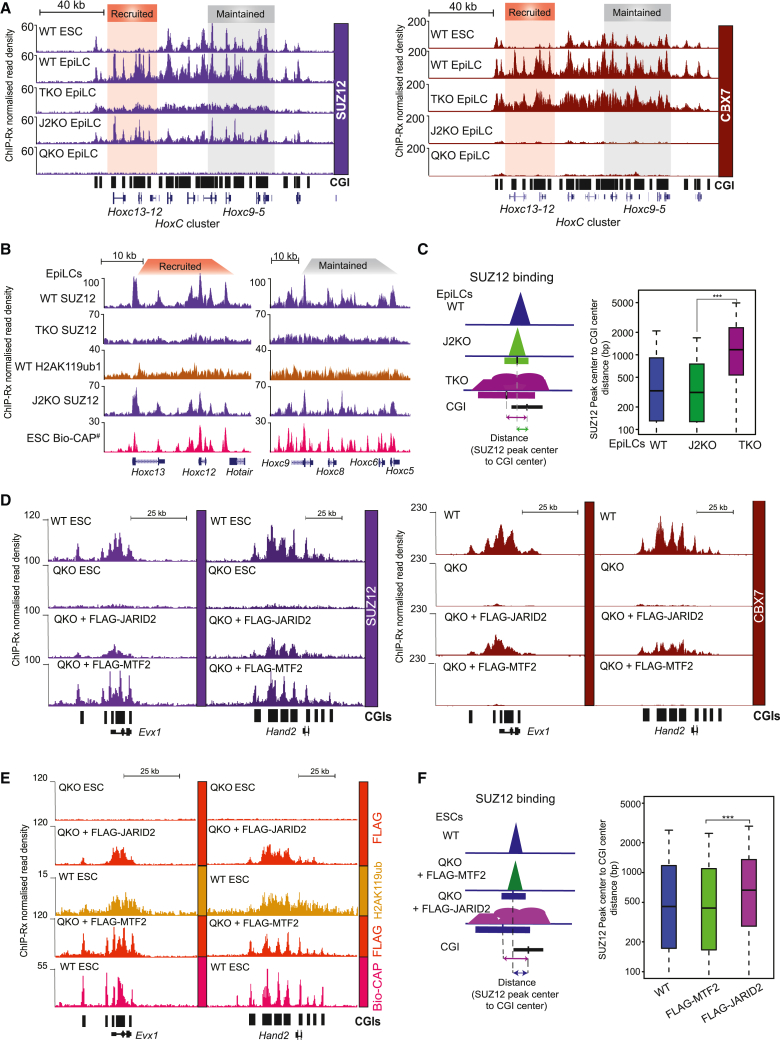
Contrasting PRC2.1 and PRC2.2 binding profiles consistent with independent recruitment mechanisms (A) Genome browser tracks showing SUZ12 and CBX7 ChIP-Rx binding in the relevant cells at the *HoxC* locus. The red region represents recruited genes, whereas the gray region represents a group of maintained genes. (B) Genome browser tracks of the indicated antibodies in the indicated cell lines. Bio-CAP tracks generated on wild-type ESCs, taken from GSE43512.^65^ (C) Left: schematic of assay design. Right: boxplot representing the distance between the SUZ12 peak center of WT, J2KO, and TKO EpiLCs and the center of the CGIs. ^∗∗∗^p value < 0.001. (D) Genome browser tracks of the indicated antibodies in the indicated cell lines at the extended *HoxC* locus. (E) Genome browser tracks of the indicated antibodies in the indicated cell lines, as well as Bio-CAP, the extended *HoxC* locus. (F) Boxplot representing the distance between the center of SUZ12 peaks in WT, and in QKO + FLAG-MTF2 or QKO + FLAG-JARID2. See also Figure S5.

Since PRC2.1 and PRC2.2 bind to target genes in ESCs via independent mechanisms,^16^^,^^20^^,^^24^^,^^48^^,^^50^^,^^64^ we sought to more closely explore their respective *de novo* recruitment profiles during ESC-EpiLC differentiation. We carefully monitored the recruitment profiles of PRC2.1 in the absence of PRC2.2 (J2KO) and separately monitored PRC2.2 in the absence of PRC2.1 (TKO). This revealed strikingly different binding profiles for each subcomplex (Figures 5A, S5A, and S5B). We first focused on PRC2 binding across the extended *HoxC* locus, which includes a region of *de novo* PRC2.1/PRC2.2 recruitment (spanning *Hoxc12-13*; red highlighted region) and another nearby region (spanning *Hoxc5-9*; gray highlighted region) where PRC2.1/PRC2.2 is maintained between ESCs and EpiLCs (Figures 5A, S5A, and S5B). In cells lacking PRC2.1 (TKO), SUZ12 was recruited in a broad, diffuse profile, whereas in cells lacking PRC2.2 (J2KO), SUZ12 was recruited in distinct peak-like profiles (Figure 5A). The broad profiles of SUZ12 in TKO EpiLCs mirrored JARID2 and CBX7 binding, as well as the broad and diffuse profiles of H2AK119ub1 (Figures 5A and S5A). By contrast, the distinct sharp peak profiles of SUZ12 in J2KO EpiLCs resembled MTF2 binding and mirrored the presence of CGIs (Figures 5A and S5B). Taken together, these distinct binding profiles of the PRC2 subcomplexes support previous evidence that PRC2.1 recruitment is dependent on CGIs, whereas PRC2.2 recruitment is dependent on H2AK119ub1.^20^^,^^23^^,^^28^^,^^47^^,^^48^

We next directly compared SUZ12 binding in J2KO and TKO cells with H2AK119ub1 profiles and with Bio-CAP-seq, a method used for capturing non-methylated CpG-rich DNA^65^ (Figure 5B). This confirmed that in EpiLCs lacking PRC2.2 (J2KO), PRC2.1 consistently aligned precisely on CGIs. On the other hand, in cells lacking PRC2.1 (TKO), PRC2.2 tracked with H2AK119ub1 (Figures 5B, S5A, and S5B). Importantly, these data replicated across several published datasets,^20^^,^^21^ and the merged binding profiles of SUZ12 from TKO and J2KO reflect WT profiles (Figures S5C and S5D). To further explore PRC2.1 binding specificity to CGIs, we compared the distance between the CGI center and SUZ12 peak center in WT EpiLCs and cells lacking either PRC2.2 (J2KO) or PRC2.1 (TKO) (Figure 5C). The distance was greater in cells lacking PRC2.1 (TKO), compared with cells lacking PRC2.2 (J2KO). We also examined the correlation between PRC2 binding, Bio-CAP, and H2AK119ub1 across all Polycomb contexts and plotted them into a hierarchical clustering heatmap. This revealed that the CBX7 and “merged” SUZ12 profiles, as well as the SUZ12, MTF2, and JARID2 ChIP-Rx of WT EpiLCs, all enriched in the same cluster (Figure S5E, blue box). This analysis further confirmed a positive correlation between PRC2.1 (MTF2 and SUZ12 in J2KO ESCs) and Bio-CAP (Figure S5E, green box) and PRC2.2 (JARID2 and SUZ12) and CBX7 in TKOs with H2AK119ub1 (Figure S5E, purple box). Taken together, these analyses highlight a distinct shift in overall binding profiles depending on whether PRC2.1 or PRC2.2 is present.

Importantly, we corroborated these results using our exogenous *de novo* recruitment assay in QKO ESCs. Strikingly, exogenous FLAG-MTF2 recruited PRC2.1 in sharp peak-like binding profiles that overlapped with Bio-CAP, whereas exogenous FLAG-JARID2 recruited PRC2.2 and CBX7-cPRC1 to broad profiles, mirroring H2AK119ub1 at several independent Polycomb target genes (Figures 5D and 5E). Furthermore, the distance between the center of a CGI and SUZ12 peak was greater in FLAG-JARID2 compared with FLAG-MTF2 expressing ESCs (Figure 5F).

Taken together, these complementary systems establish that MTF2 directs PRC2.1 specifically to CGIs, whereas JARID2 is essential for directing PRC2.2 and CBX7-cPRC1 to broader regions, which mirror H2AK119ub1 deposition (Figure S5F).

### DNA and histone modification binding activities of MTF2 and JARID2 facilitate PRC2.1 and PRC2.2 chromatin binding

**Figure 6:**
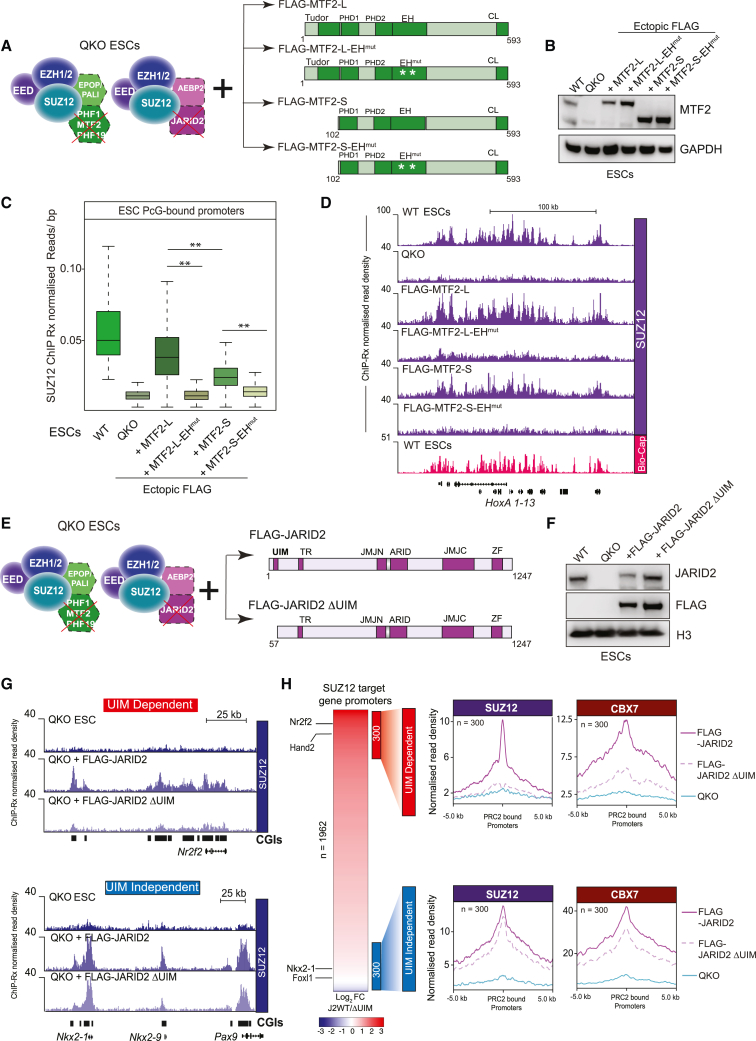
DNA and histone modification binding activities of MTF2 and JARID2 facilitate the respective chromatin binding of PRC2.1 and PRC2.2 (A) Schematic of wild-type or mutant MTF2 rescue strategy. (B) Western blot analyses using the indicated antibodies of total protein extracts in the indicated ESC lines. (C) Boxplot of SUZ12 ChIP-Rx read counts in the indicated ESC lines at all PRC2-bound promoters. ^∗∗^p value < 0.01. (D) Genome browser tracks showing SUZ12 ChIP-Rx profile on the *HoxA* locus in the indicated cell lines. Note the y axis values are adjusted to facilitate the visualization of the tracks. (E) Schematic of wild-type or truncated JARID2 rescue strategy. (F) Western blot analyses using the indicated antibodies on total protein extracts in the indicated ESC lines. (G) Genome browser tracks showing SUZ12 ChIP-Rx profile in the indicated cell lines at representative UIM-dependent (top) and UIM-independent (bottom) gene loci. (H) Left: heatmap representing the fold change of SUZ12 binding at PRC2 target promoters in QKO + JARID2-WT or QKO + JARID2-ΔUIM. Right: average plots showing ChIP-Rx normalized read densities for SUZ12 and CBX7 at the UIM-dependent (n = 300) and UIM-independent (n = 300) regions, in QKO and JARID2 rescue ESCs. See also Figure S6.

Next, we used our ectopic expression system to dissect the functional domains within the MTF2 and JARID2 proteins, which are necessary for the independent recruitment of PRC2.1 and PRC2.2, respectively. MTF2 is a multi-domain protein, containing a Tudor domain reported to bind *in vitro* to the H3K36me2/3 posttranslational modifications, and to a lesser extent to H3K27me3,^51^^,^^52^^,^^53^^,^^54^ and an EH domain capable of binding DNA with high affinity.^47^^,^^48^^,^^50^ The short isoform of MTF2 naturally lacks a Tudor domain, thereby facilitating a comparison between MTF2 with and without this domain. To disrupt MTF2-EH domain function, we introduced charge-swap mutations to two positively charged residues in the helix 3 region and to two conserved lysine residues in the wing 1 region (Figure S6A). To delineate the contributions of the MTF2-EH and MTF2-Tudor domains for targeting PRC2.1 to chromatin, we rescued QKO ESCs with FLAG-MTF2-L, FLAG-MTF2-L with the EH domain mutated (FLAG-MTF2-L-EH^mut^), FLAG-MTF2-S (lacking Tudor domain), and MTF2-S with the EH domain mutated (FLAG-MTF2-S-EH^mut^) (Figure 6A). Exogenous expression of each MTF2 protein was achieved to comparable levels, and initial ChIP-qPCR analyses of SUZ12, FLAG, and EPOP in the rescued QKO ESC lines revealed that expression of FLAG-MTF2-L rescued PRC2.1 binding at Polycomb target genes (Figures 6B and S6B). Strikingly, the disruption of the EH domain in either MTF2-L or MTF2-S was sufficient to completely deplete PRC2.1 recruitment (Figures 6C, 6D, S6B, and S6C), consistent with previous findings.^47^^,^^48^^,^^50^ Interestingly, exogenous expression of FLAG-MTF2-S, which lacks the Tudor domain, was capable of partially rescuing PRC2.1 binding, albeit to a lesser extent than FLAG-MTF2-L (Figures 6C, 6D, S6B, and S6C). However, while the PRC2.1 binding profile in the MTF2-S line still correlated with Bio-CAP peaks at CGIs (Figure 6D), its reduced binding at these sites was accompanied by moderate increases elsewhere in the genome (Figure S6D). Taken together, while these data support previous reports that the MTF2-EH domain is the primary mediator of PRC2.1 binding at CGIs, they also establish that the MTF2-Tudor domain further stabilizes PRC2.1 at these sites.

Next, to evaluate the contribution of the JARID2-UIM to the recruitment of PRC2.2 to Polycomb target genes, we ectopically expressed either WT FLAG-JARID2 or a truncated version, lacking the N-terminal region containing the UIM, in QKO ESCs (Figures 6E–6G). ChIP-Rx analysis of SUZ12 revealed that exogenous expression of FLAG-JARID2 partially rescued overall PRC2 binding at Polycomb target genes (Figures S6E and S6F). We identified a subset of target genes, including *Nr2f2* and *Hand2*, which was JARID2-UIM dependent, while another subcohort of target genes, including *Pax9 and Nkx2-1*, was JARID2-UIM independent (Figure 6G). We next ranked all PRC2-bound promoters based on the difference in SUZ12 binding between FLAG-JARID2 and FLAG-JARID2-Δ UIM expressing ESCs (Figure 6H, left). Average plots of SUZ12 and CBX7 binding at the top 300 “UIM-dependent” and “UIM-independent” Polycomb target genes confirmed that the ability of PRC2.2 to recruit CBX7-cPRC1 was dependent on the JARID2-UIM at the UIM-dependent sites (Figures 6H, right and S6G). The UIM-dependent sites had lower enrichments of H2AK119ub1, H3K27me3, MTF2, and JARID2 in WT ESCs, compared with the UIM-independent sites (Figure S6H). This indicates that the UIM-dependent sites are weaker Polycomb target genes and therefore perhaps more susceptible to changes in stabilizing interactions. We anticipate that additional interactions such as those mediated by AEBP2 may contribute to PRC2.2 binding at UIM-independent sites in the absence of the JARID2-UIM.

### JARID2 promotes 3D chromatin looping through CBX7-cPRC1 at Polycomb target genes

**Figure 7:**
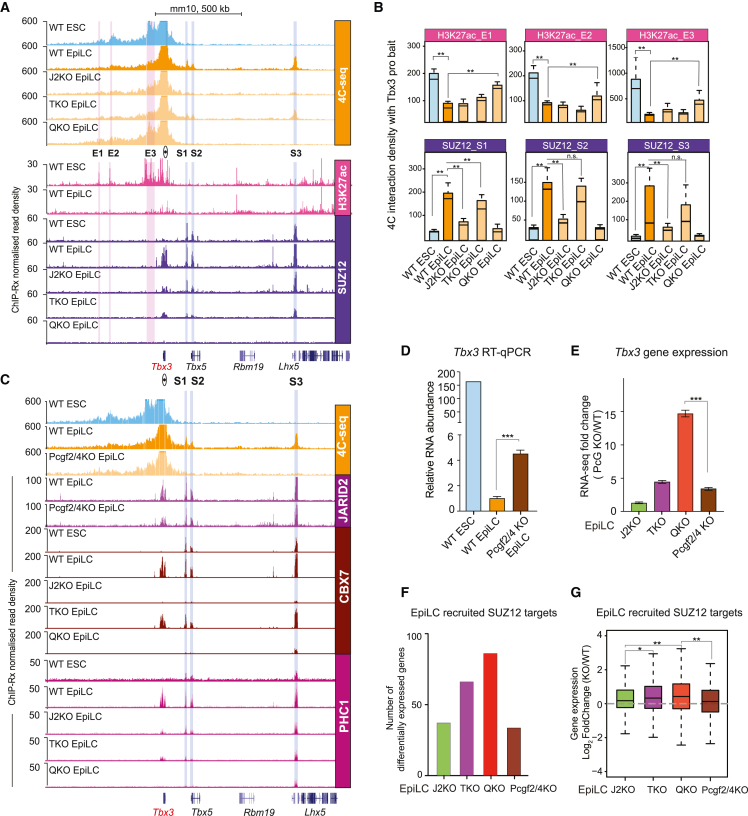
PRC2.1-deposited H3K27me3 and PRC2.2-JARID2-recruited CBX7-cPRC1 cooperate to mediate Polycomb target repression (A) Genome browser tracks showing 4C-seq analyses of the indicated cell lines using the *Tbx3* gene promoter as the viewpoint bait. SUZ12 and H3K27ac ChIP-Rx profiles are shown below. SUZ12-bound sites (S1, S2, and S3) are highlighted in blue, while the H3K27ac-enriched enhancers (E1, E2, and E3) are highlighted in pink. (B) Boxplots representing the 4C-seq densities at S1, S2, and S3 and E1, E2, and E3 in the indicated cell lines. ^∗∗^p value < 0.01. (C) Genome browser tracks showing 4C-seq analyses of the indicated cell lines using the *Tbx3* gene promoter as the viewpoint bait. ChIP-Rx profiles of the indicated antibodies in the relevant cell lines are also shown. (D) Relative mRNA abundance of *Tbx3* in WT ESCs, and WT and *Pcgf2/4* KO EpiLCs. Error bars represent SD (n = 3). (E) Bar plots representing the fold change of *Tbx3* expression between WT and PcG mutant EpiLCs. ^∗∗∗^p value < 0.001. Error bars represent SD (n = 3). (F) Bar plots representing the number of DESeq2-identified differentially expressed recruited Polycomb target genes in the indicated EpiLCs (n = 3). (G) Boxplots showing RNA-seq log_2_-fold change of WT compared with the respective KO EpiLCs. The gray dashed line indicates the y axis at 0. ^∗^p value < 0.05 and ^∗∗^p value < 0.01. Error bars represent SD (n = 3). See also Figure S7.

To explore the potential contributions of PRC2.1 and PRC2.2 to 3D chromatin interactions at a *de novo* Polycomb target gene during ESC-EpiLC differentiation, we performed circular chromosome conformation capture sequencing (4C-seq) at the *Tbx3* promoter in WT ESCs, WT EpiLCs, and EpiLCs lacking key PRC2 accessory proteins (Figure 7A). In WT ESCs, the interactions that were lost during the transition to EpiLCs were located at H3K27ac-enriched, ESC-specific enhancers (e.g., E1, E2, and E3) (Figures 7A, 7B, and S7A). The p300 acetyltransferase and OCT4 were previously shown to bind to these three enhancer elements in ESCs^66^ and are displaced upon differentiation to EpiLCs (Figure S7A). The sites that gained *de novo* interactions with the *Tbx3* promoter in WT EpiLCs were pre-bound distal SUZ12-enriched sites (e.g., S1, S2, and S3), which co-localized with JARID2, MTF2, and H3K27me3, and correspond to the promoters of the nearby *Tbx5* and *Lhx5* genes (Figures 7A and S7B). Both *Tbx5* and *Lhx5* are Polycomb repressed in both ESCs and EpiLCs and therefore classed among the “maintained” group of PRC2-bound genes (Figures 1B and S7C). Loss of PRC2.1, PRC2.2, or both caused concurrent increases in *Tbx3* expression and associated H3K27ac deposition at recruited target sites (Figures S7C and S7D). Strikingly, the *de novo* interactions with the *Tbx3* promoter did not occur in J2KO and QKO EpiLCs but were still able to form in TKO EpiLCs (Figures 7A and 7B). Importantly, JARID2 binding was maintained at these sites in TKO EpiLCs (Figures 7C and S7B). We next confirmed that cPRC1 subunits PHC1 and CBX7 are precisely co-localized with the PRC2 subcomplexes at S1, S2, and S3 regions in WT ESCs and EpiLCs, and CBX7 is not bound in J2KO and QKO cells (Figure 7C). We performed 4C-seq in *Pcgf2/4* KO EpiLCs (Figure 7C), in which the majority of CBX7 localization is lost (Figure S7E). The interactions between the *Tbx3* promoter and the pre-bound Polycomb promoter regions (S1, S2, and S3) were lost in the absence of cPRC1 (Figure 7C), despite unaffected JARID2 binding, and this correlated with impaired repression of *Tbx3* during EpiLC differentiation (Figures 7D and 7E). Taken together, these data suggest that the proper repression of this *de novo* Polycomb target gene during differentiation is at least partially dependent on the activities of JARID2 to promote the focal recruitment of CBX7-cPRC1, which in turn is required to establish long-range chromatin interactions to either create or expand a Polycomb-repressed domain.

Finally, to explore the consequences of impaired recruitment of the PRC2 subcomplexes on Polycomb target gene expression, we compared global RNA-seq of WT and PRC2-mutant EpiLCs (Figure S7F). The total number of differentially expressed direct target genes was greater in TKO EpiLCs, compared with J2KO EpiLCs (Figure S7F). While the number of differentially expressed genes did not vary much between TKO and QKO EpiLCs (Figure S7F), the degree to which genes such as *Tbx3* were upregulated in QKO compared with TKO and J2KO was significantly greater (Figures 7E–7G). We found that the level of impaired gene repression in cells lacking *Pcgf2/4* was similar to that for loss of JARID2, while there was a significantly greater increase in the impaired repression in QKO and TKO (Figures 7F and 7G).

Taken together, these data support a model whereby two distinct axes, directed by PRC2.1 and PRC2.2, combine for faithful repression of Polycomb target genes during differentiation. This occurs via a combination of PRC2.1-mediated broad H3K27me3 depositions and JARID2-dependent CBX7-cPRC1-mediated 3D looping, both of which contribute to the transition of the chromatin and transcriptional landscape.

## Discussion

It has been unclear why two independent PRC2 subcomplexes, PRC2.1 and PRC2.2, have persisted throughout evolution. Here, through genetic KO and replacement of key specific subunits, we discover independent functions of PRC2.1 and PRC2.2 in mediating Polycomb target gene repression. PRC2.1 has a specialized role in promoting the majority of H3K27me3 deposition, which promotes CBX2/4-cPRC1 recruitment, whereas PRC2.2 component JARID2 drives recruitment of CBX7-cPRC1. Furthermore, we define how PRC2.1 and PRC2.2 are independently recruited to Polycomb target genes via the DNA and chromatin-binding abilities of their specific subunits MTF2 and JARID2. The combination of their respective actions in promoting H3K27me3 deposition, cPRC1 recruitment, and in turn 3D chromatin looping, ultimately establishes stable Polycomb domains and gene repression during a cell-fate transition.

### PRC2.1 and PRC2.2 are co-recruited during pluripotent-state transition to promote repression of *de novo* Polycomb target genes

We establish that PRC2.1 and PRC2.2 are co-displaced from and co-recruited to Polycomb target genes during ESC-EpiLC differentiation. While it is possible that PRC2.1 and PRC2.2 regulate divergent sets of target genes during differentiation, our focus here on direct Polycomb target genes does not provide supporting evidence. Notably, the distinct recruitment mechanisms of PRC2.1 and PRC2.2 via CpG and H2AK119ub1 binding, respectively, coupled with more restricted expression of key accessory proteins, could provide avenues for distinct target gene regulation in different cellular contexts and other stages during development. Despite this, the mouse KO phenotypes of *Jarid2* or *Mtf2* cause prenatal lethality—indicating both are required for early development.^3^^,^^67^^,^^68^^,^^69^^,^^70^ However, in the absence of a mouse model lacking JARID2 and all three Polycomb-like proteins, it’s not yet known if the combined loss of PRC2.1 and PRC2.2 functions would have a more severe developmental phenotype.

While PRC2.1 and PRC2.2 have been reported to be recruited to chromatin via alternative mechanisms,^20^^,^^23^^,^^24^^,^^28^^,^^46^^,^^47^^,^^48^^,^^50^ we extend this by showing PRC2.1 binds precisely at CGIs, forming narrow peak-like profiles, whereas PRC2.2 binds in broader profiles, mirroring H2AK119ub1 deposition.

### JARID2 drives CBX7-cPRC1 recruitment while MTF2-PRC2.1 drives CBX2/4-cPRC1 recruitment

The prevailing model of cPRC1 recruitment involves its association with target genes through an affinity of chromodomain-containing CBX subunits for H3K27me3, and this facilitates subsequent Polycomb domain formation and compaction.^29^^,^^30^^,^^31^^,^^32^ However, there are five cPRC1-associated CBX proteins (CBX2/4/6/7/8) expressed in mammalian cells. While each of their chromodomains have varying affinities for H3K27me3, all display a lower affinity, compared with their homologous *Drosophila* subunit dPc.^71^ Although we show that CBX7 can immunoprecipitate vPRC1-PCGF1 and -PCGF6 proteins, their respective stoichiometries with CBX7 are more than 20-fold lower compared with cPRC1 member, PHC1. This, together with changes in PHC1 occupancy upon modulation of JARID2 levels, implies that CBX7 is recruited together with PHC1 in the context of cPRC1. Taken together, our data suggest that while CBX7-cPRC1 localization to target genes would appear to require at least low levels of H3K27me3, the extent of its recruitment is not modulated by changes in the level of H3K27me3 enrichment.

Interestingly, mammalian CBX chromodomains can bind to DNA as well as H3K27me3.^72^^,^^73^^,^^74^ Similarly, the C terminus of JARID2 has been reported to have affinity for DNA.^75^ While the exact mechanism of JARID2-dependent CBX7 recruitment is still unclear, a recent cross-linked IP mass spectrometry experiment suggested JARID2 and CBX7 are in close proximity at target sites.^33^ We speculate that the recruitment mechanism could involve the ability of CBX7 to interact with DNA and histones, in addition to its ability to bind to H3K27me3. Supporting this, cPRC1 has been reported to associate with target genes in the presence of PRC2, but absence of H3K27me3, in *Drosophila* larval tissues.^76^ Taken together with our data, this raises the possibility of H3K27me3-independent associations of cPRC1 with chromatin. In fact, an interesting possibility is that JARID2 promotes CBX7-cPRC1 recruitment as part of the less enzymatically active EZH1-PRC2.

### Deciphering the DNA and histone-binding domains required for PRC2.1 and PRC2.2 recruitment

Here, using an exogenous rescue assay, we establish that the JARID2-UIM is not required for PRC2.2 binding at approximately half of all PRC2.2 target genes in ESCs. This subcohort of Polycomb target genes that do not require the JARID2-UIM is associated with higher enrichment of Polycomb proteins. Therefore, we speculate that the presence of AEBP2 provides sufficient interaction for PRC2.2 to bind at these loci. Additionally, the JARID2-ARID and -zinc finger DNA-binding domains may also play a role.^75^^,^^77^^,^^78^

We also decipher the contributions of the MTF2-EH and MTF2-Tudor domains for targeting PRC2.1 to target genes. We find that that the EH domain is essential for targeting PRC2.1 to all target genes in ESCs, supporting previous studies.^48^ Intriguingly, a direct comparison of the naturally occurring short and long MTF2 isoforms revealed that the Tudor domain also contributes to overall levels of PRC2.1 binding at target genes. However, its loss did not impair the specificity of PRC2.1 binding to CGIs. Furthermore, the short isoform lacking the Tudor domain had moderately increased binding at regions outside of Polycomb target genes. This suggests that the Tudor domain contributes to stabilizing PRC2.1 at CGIs. Potentially, it could engage with or “sample” H3K36me2 and/or H3K36me3 elsewhere throughout the genome and function to direct PRC2.1 away from these regions.

Overall, we have uncovered independent functions for PRC2.1 and PRC2.2, which reshape our understanding of the hierarchical recruitment model for Polycomb complexes. The prevailing model implicates H3K27me3 as the sole recruiting factor for CBX proteins in cPRC1. Instead, our results place JARID2 at the forefront of CBX7-cPRC1 recruitment. In addition, we elucidated the mechanisms through which the distinct PRC2.1 and PRC2.2 subcomplexes bind on chromatin and contribute to establish *de novo* Polycomb-mediated repression during the transition from naive to primed pluripotency. Together, these results reshape our understanding of the mechanisms governing Polycomb system function.

### Limitations of the study

Although the data provided in this paper show that JARID2 is essential for CBX7-cPRC1 association with Polycomb target genes, the precise mechanism remains elusive. While we establish that fellow PRC2.2 component AEBP2 does not have a role, further dissection of the domains within JARID2 will be necessary to evaluate how it exerts its control on CBX7-cPRC1. In addition, further studies are needed to compare the binding dependencies of all CBX proteins (CBX2/4/6/8) of cPRC1 and to dissect their likely divergent mechanisms of chromatin targeting. Finally, while we established that PRC2.1- and PRC2.2-specific accessory proteins drive the recruitment of different forms of cPRC1 to chromatin in pluripotent ESCs and pre-gastrulation EpiLCs, it will be important to further evaluate the biological significance of these findings in additional cellular contexts, including models of development and disease.

## STAR★Methods

### Key resources table

**Table:** 

REAGENT or RESOURCE	SOURCE	IDENTIFIER
**Antibodies**		

H3K27me3; ChIP	Cell Signaling	Cat#9733 (C36B11); RRID: AB_2616029
H2K119ub1; ChIP, WB	Cell Signaling	Cat#8240 (D27C4); RRID: AB_10891618
H3K27me3; WB	Active motif	Cat#61017; RRID: AB_2614987
H3K27ac; ChIP	Abcam	Cat#ab4729; RRID: AB_2118291
SUZ12; ChIP, WB	Cell Signaling	Cat#3737 (D39F6); RRID: AB_2196850
JARID2; ChIP, WB	Cell Signaling	Cat#13594 (D6M9X); RRID: AB_2798269
MTF2; ChIP, WB	Peprotech	Cat#16208-1-AP; RRID: AB_2147370
CBX7; ChIP, WB	Abcam	Cat#ab21873; RRID: AB_726005
CBX7; ChIP, IP-MS	Millipore	Cat#07-981; RRID:AB_10807034
CBX2; ChIP, WB	Cell Signaling	Cat#18687
CBX4; ChIP, WB	Cell Signaling	Cat#44268; RRID:AB_2799261
PHC1; ChIP, WB	Cell Signaling	Cat#13768; RRID:AB_2716803
FLAG; ChIP, WB	Gift from Dr. Claudio Ciferri	N/A
IgG; ChIP	Merck	Cat#12371; RRID: AB_145840
H3; WB	Abcam	Cat#ab1791; RRID: AB_302613
IRDye 800CW Goat anti-Mouse IgG; WB	LI-COR	Cat#925-32210; RRID: AB_2687825
IRDye 800CW Goat anti-Rabbit IgG; WB	LI-COR	Cat#925-32211; RRID: AB_2651127
IRDye 680LT Goat anti-Mouse IgG; WB	LI-COR	Cat#925-68020; RRID: AB_2687826
IRDye 680LT Goat anti-Rabbit IgG; WB	LI-COR	Cat#925-68021; RRID: AB_2713919

**Chemicals, peptides, and recombinant proteins**		

2i-PD0325901	CAYMAN	Cat#13034-10
2i- CHIR99021	CAYMAN	Cat#13122-10
bFGF Recombinant Human Protein	Gibco	Cat#13256029
Activin A	Peprotech	Cat#120-14
Fibronectin	Millipore	Cat#FC010
Tazemetostat (EPZ-6438)	Selleck Chemicals	Cat#S7128
Formaldehyde	Sigma-Aldrich	Cat#252549
Triton X-100	Sigma-Aldrich	Cat#T8787
IGEPAL CA-630	Sigma-Aldrich	Cat#I8896
Phenol – chloroform – isoamyl alcohol mixture	Sigma-Aldrich	Cat#77617
Proteinase K	Sigma-Aldrich	Cat#P2308
RNase A	Thermo Fisher Scientific	Cat#EN0531

**Critical commercial assays**		

NDiff227 medium	Takara	Cat#Y40002
GMEM medium	Sigma-Aldrich	Cat#G5154
AMPure beads	Beckman Counter	Cat#A63881
Protein-A-Dyna Beads	Thermo Fisher Scientific	Cat#10001D
Protein-G-Dyna Beads	Thermo Fisher Scientific	Cat#10003D
RNeasy Mini Kit	Qiagen	Cat#74104
RNase-Free DNase Set	Qiagen	Cat#79254
T4 DNA ligase	New England Biolabs	Cat#M0202M
T4 DNA ligase buffer	New England Biolabs	Cat#B0202S
High Sensitivity D1000 Reagents	Agilent	Cat#5067-5585
High Sensitivity D1000 ScreenTape	Agilent	Cat#5067-5584
Q5 Site-Directed Mutagenesis Kit	New England Biolabs	Cat#E0554
Luna Universal qPCR Master Mix	New England Biolabs	Cat#M3003E
NEBNext Ultra II DNA Library Kit for Illumina	New England Biolabs	Cat#E7645
NEBNext Poly(A) mRNA Magnetic Isolation Module	New England Biolabs	Cat#E7490
NEBNext Ultra RNA Library Prep Kit	New England Biolabs	Cat#E7770
Expand Long Template PCR System	Roche	Cat#11759060001

**Deposited data**		

ChIP-seq	This paper	GEO:GSE199530
RNA-seq	This paper	GEO:GSE199530
4C-seq	This paper	GEO:GSE199530
Original western blot and gel images	This paper	Mendeley Data: https://doi.org/10.17632/t969jsj3t7.1
ESC Bio-CAP	Long et al.^65^	GEO:GSE43512
ESC ChIP-seq	Healy et al.^20^	GEO:GSE127121
ESC ChIP-seq	Hojfeldt et al.^21^	GEO:GSE127804
OCT4 and P300 ChIP-seq	Buecker et al.^66^	GEO:GSE56138

**Experimental models: Cell lines**		

J2WT mESC	Fisher lab	Landeira et al.^79^
JARID2 KO mESC	Fisher lab	Landeira et al.^79^
PclWT mESC	Koseki lab	N/A
PHF1, MTF2, PHF19 TKO mESC	Koseki lab	N/A
JARID2,PHF1, MTF2, PHF19 QKO mESC	Bracken lab	Healy et al.^20^
Pcl JARID2 KO ESC	Bracken lab	Healy et al.^20^
QKO+res MTF2-L mESC	This paper	N/A
QKO+res MTF2-S mESC	This paper	N/A
QKO+res MTF2-L-EHmut mESC	This paper	N/A
QKO+res MTF2-S-EHmut mESC	This paper	N/A
QKO+res JARID2-Full lenth (FL) mESC	This paper	N/A
QKO+res JARID2-ΔUIM	This paper	N/A
Ezh1/2 dKO mESC	Pasini lab	Lavarone et al.^63^
Ezh1 KO/Ezh2 Y726D mESC	Pasini lab	Lavarone et al.^63^

**Software and algorithms**		

Botwie2, v2.3.4.3	Langmead and Salzberg^80^	http://bowtie-bio.sourceforge.net/bowtie2/index.shtml
Ensembl, GRCm38.101	Yates et al.^81^	https://www.ensembl.org/index.html
Fastqc, v0.11.9	Andrews^82^	http://www.bioinformatics.babraham.ac.uk/projects/fastqc/
macs2, v2.2.7.1	Feng et al.^83^	https://github.com/macs3-project/MACS/releases/tag/v2.2.7.1
samtools, v1.9	Li et al.^84^	http://www.htslib.org/
bedtools, v2.27.1	Quinlan and Hall^85^	https://bedtools.readthedocs.io/en/latest/
deeptools, v3.3.0	Ramirez et al.^86^	https://deeptools.readthedocs.io/en/develop/
Picard tools	Broad Institute	http://broadinstitute.github.io/picard/
STAR, v2.7.1a	Dobin et al.^87^	https://github.com/alexdobin/STAR
DESeq2, v1.22.1	Love et al.^88^	https://bioconductor.org/packages/release/bioc/html/DESeq2.html
featureCounts, v1.6.4	Liao et al.^89^	https://rdocumentation.org/packages/Rsubread/versions/1.16.1
R:pheatmap,v1.0.12	Klode^90^, R package, 2012	https://cran.r-project.org/web/packages/pheatmap/index.html
R:ggplot2, v3.3.3	Wickham, R package, 2011^91^	https://cran.r-project.org/web/packages/ggplot2/index.html
pipe4C	Krijger et al.^92^	https://github.com/deLaatLab/pipe4C
intervene	Khan and Mathelier^93^	https://intervene.readthedocs.io/
MaxQuant 1.6.0.1	Cox and Mann^94^	https://www.maxquant.org/
R v3.5.1	R Core Team	https://cran.r-project.org/

**Other**		

NextSeq 500/550 High Output Kit v2.5 (75 Cycles)	Illumina	Cat# 20024906

### Resource availability

#### Lead contact

Further information and request for resources and reagents should be directed to and will be fulfilled by the lead contact, Adrian Bracken (adrian.bracken@tcd.ie).

#### Materials availability

Commercially available reagents are listed in the key resources table. All plasmids or cell lines generated in this study are available on request.

### Experimental model and subject details

#### Cell culture

Mouse embryonic stem cells (ESCs) were grown on gelatinized culture dishes in GMEM (Sigma) supplemented with 20% FBS (Gibco), 100U/mL Penicillin-Streptomycin (Gibco), 50 μM β-mercaptoethanol (Sigma), 1:100 GlutaMax, 1:100 non-essential amino acids (Gibco), 1mM sodium pyruvate (Gibco), 1:500 homemade leukaemia inhibitory factor (LIF), and 2i components; 3 μM GSK inhibitor CHIRON99021 (Cayman) and 1 μM MEK inhibitor PD0325901 (Cayman).The ESCs were spited and changed medium every 2 days. For EpiLC differentiation experiments, the dishes were coated with 16 μg/ml fibronectin for 2∼3 hours. 2 million ESCs were washed three times with PBS and seeded on the fibronectin coated dish in NDiff227 medium (Takara) supplemented with 20 ng/ml Activin A (Peprotech), 12 ng/ml bFGF (Gibco) and 1% Knockout Serum Replacement (KSR, Thermo) for 2 days. The EpiLC culture medium were changed daily. Human NTERA2 embryonic carcinoma cells (NT2) for ChIP-Rx spike-in were cultured in DMEM supplemented with 10% FBS (GIBCO), 100 U/mL penicillin (GIBCO) and 100U/mL streptomycin (GIBCO).

### Method details

#### Exogenous expression in mESCs

For rescue experiments, pCAG or pLenti vectors encoding FLAG-HA-tagged human JARID2 or MTF2 constructs were generated by site-directed mutagenesis (Q5 SDM kit, New England Biolabs). pCAG vectors were transfected into knockout ESCs using Lipofectamine 2000 (Thermo Fisher). Stable clones were derived through puromycin-selection. Single clones were expanded, and stable integration of the construct was screened by western blot. The pLenti venti vector was used to make lenti-virus. This was transduced into knockout ESCs. Stable clones were derived through puromycin-selection and stable integration of the construct was screened by western blot analysis.

#### Inhibition of PRC2 histone methyltransferase activity

E14 mouse ESCs were treated with high concentration of (10 μM) of Tazemetostat for 7 days. Cells were harvested for western blot analyses at days 2, 5, and 7, and for ChIP-qPCR analyses at day 7.

#### Preparation of nuclear lysates and western blotting

Cells were scraped down to collect them, washed three times in PBS and resuspended in ice cold nuclear extract buffer (10 mM Tris pH 8.0, 100 mM NaCl, 2 mM MgCl2, 0.3 M Sucrose, 0.25% NP40, 1mM PMSF, 2 mg/mL Aprotinin, 1 mg/mL Leupeptin). Lysate was then passed through a tight dounce six times and centrifuged for 15 minutes at 200rpm at 4°C to isolate nuclei. Nuclei were then lysed in ice cold High Salt buffer (50 mM Tris-HCl, pH 7.2, 300mM NaCl, 0.5% (v/v) NP-40, 1mM EDTA pH7.4, 2 mg/mL Aprotinin, 1 mg/mL Leupeptin, 1mM PMSF). Cells were then sonicated and incubated for 20 minutes at 4 °C while rotating to ensure sufficient lysis. The lysates were then clarified at 14,000rpm at 4 °C for 25 mins. Nuclear lysates were then separated on SDS-PAGE gels and transferred to nitrocellulose membranes. Membranes were subsequently probed using the relevant primary (overnight at 4 °C) and secondary (1 hour at room temperature) antibodies. Relative proteins levels were then determined by chemiluminescence or fluorescence-based approaches on an Odyssey LiCOR Fc imaging system.

#### FLAG immunoprecipitation

Whole cell lysates of QKO mESCs expressing Flag-tagged JARID2 and Flag-tagged MTF2 constructs were prepared in High Salt buffer (50 mM Tris-HCl pH7.2, 300 mM NaCl, 0.5% (v/v) NP-40, 1 mM EDTA pH7.4, 2 μg/mL Aprotinin, 1 μg/mL Leupeptin, 1mM PMSF), sonicated 3 x 10 sec pulses and subjected to tight dounce 20 times. The lysates were rotated at 4°C for 20 mins. Before being diluted with No Salt Buffer (50 mM Tris-HCl, 0.5% (v/v) NP-40, 1 mM EDTA pH7.4, 2 μg/mL Aprotinin, 1 μg/mL Leupeptin, 1mM PMSF). The lysates were incubated with 20 μL anti-FLAG M2 beads (Genentech) overnight with rotation at 4 °C, in the presence of 250 U/mL Benzonase nuclease. Beads were washed 5 times with wash buffer (1:1 dilution of, high salt buffer: no salt buffer). Precipitated proteins were eluted from the beads by addition of 60 μL 0.5 mg/mL FLAG-peptide (sequence: DYKDDDDK), while shaking at 25°C for 30 min.

#### Endogenous CBX7 immunoprecipitations

Cells were resuspended in Buffer C (20mM HEPES pH 7.9, 0.2mM EDTA, 1.5mM MgCl_2_, 20% glycerol, 420mM NaCl, 2μg/mL Aprotinin, 1 μg/mL Leupeptin, 1mM PMSF), sonicated 3 x 15 seconds and dounced 20 times with a tight pestle. Lysates were incubated for 20 min rotating at 4 °C and clarified by centrifugation at 20,817g at 4 °C for 20 min. Lysates were dialysed for 5 hours at 4 °C against 50 volumes of Buffer C100 (20mM HEPES pH 7.9, 0.2 mM EDTA, 1.5 mM MgCl2, 20% glycerol, 125 mM KCl). Lysates were again clarified by centrifugation at 20,817g at 4 °C for 20 min. 5 μg antibody was coupled to 20μL packed Protein A beads (Sigma) by incubation in 1mL PBS (0.1% Tween-20) at 4 °C rotating overnight. Beads were collected by centrifugation at 5,440g at room temperature and washed twice in 1 mL 0.2 M Sodium Borate pH 9.0. Antibodies were then crosslinked to beads by incubation in 1mL 0.2M Sodium Borate pH 9.0 (containing 20mM dimethyl pimelimidate dihydrochloride) at room temperature rotating for 30 min. The reaction was quenched by washing beads once in 1 mL 0.2 M Ethanolamine pH 8.0 and incubating for 2 hr at room temperature rotating in 1 mL 0.2 M Ethanolamine pH 8.0. The beads were washed once in Buffer C100 and blocked for 60 minutes at 4 °C rotating in Buffer C100 (0.1mg/mL Insulin (Sigma), 0.2mg/mL Chicken egg albumin (Sigma), 0.1% (v/v) fish skin gelatin (Sigma)). Antibody-crosslinked beads were incubated with protein lysates, in the presence of 250U/mL Benzonase nuclease, at 4 °C rotating overnight and then washed 5 times in Buffer C100 (+0.02% NP-40). After the final wash, beads were resuspended in 100 μL of SDS-PAGE sample buffer. Immunoprecipitated material was eluted by boiling for 5 min with shaking before centrifuging the beads at 20,817g for 5 minutes and keeping the resulting supernatant.

#### Mass spectrometry measurements and analysis

All immunoprecipitations for mass spectrometry were performed in triplicate. After the final wash, beads were resuspended in 50 μl elution buffer (2M Urea, 100 mM Tris pH 8, 10 mM DTT) and incubated 20 min on a shaker (1300 rpm) at RT. After incubation, iodoacetamide was added to a final concentration of 50 mM, followed by 10 min shaking in the dark at RT. Partial digestion and elution from the beads was initiated by adding 0.25 mg Trypsin (Promega; V5113) for 2 hr. The supernatant containing the IP samples was collected and the beads were resuspended in 50 μL elution buffer followed by a 5 min incubation shaking at RT. Both supernatants were combined and 0.1 mg Trypsin was added followed by overnight incubation at RT. The digestion was stopped by adding TFA (final concentration 0.5%). The resulting digested samples were desalted and purified using StageTips.^95^ The peptides were eluted from StageTips with buffer B (80% acetonitrile, 0.1% formic acid), concentrated to 5 μL by SpeedVac centrifugation at room temperature, and filled up to 12 μL using buffer A (0.1% formic acid). Pulldown samples were measured on an Orbitrap Exploris (Thermo Fisher Scientific) using a gradient from 9%–32% Buffer B for 50 min followed by washes at 50% then 95% Buffer B resulting in total of 60 min data collection time. Scans were collected in data-dependent top speed mode with dynamic exclusion set at 45 s. Acquired mass spectra were analyzed with MaxQuant 1.6.0.1^94^ with default settings, and algorithms for label-free quantification and iBAQ (intensity based absolute quantification) were enabled, and by searching the mouse UniProt protein database downloaded in June 2017. All MaxQuant output was analysed with R (version 4.0.3) and enrichment analysis of the pulldowns was done with the use of the DEP package for differential enrichment analysis of proteomics data^96^ to determine significant interactors of CBX7 over IgG control.

#### RNA extraction and RT-PCR analysis

Total RNA was extracted using the Qiagen RNeasy RNA kit with DNaseI on-column treatment. Then cDNA was synthesized with High-Capacity cDNA Reverse Transcription kit (Thermo Fisher). For quantitative RT-PCR, cDNA was amplified using Luna Universal qPCR Master Mix (NEB) with specific primers on QuantStudio 3 real-time PCR systems (Applied Biosystems).

#### Chromatin immunoprecipitations

Cells were collected and washed with PBS 2x, then suspended in 10 ml PBS. Next, cells were fixed with 1% formaldehyde (Sigma) with rotation for 10 minutes, followed by quenching with glycine. Fixed cells were washed with cold PBS (with proteinase inhibitors cocktails, PIC) then lysed in SDS lysis buffer (0.5% SDS, 100mM NaCl, 50mM Tris pH 8.0, 5mM EDTA pH 8.0) pulsed PIC. The lysate was pelleted by spinning at 1400 rpm for 5 minutes at room temperature. With discarding the supernatant, the chromatin pellet was suspended in 0.33% SDS incubation buffer (0.3% SDS, 1.6% Triton X-100, 100mM NaCl, 50mM Tris pH 8.0, 5mM EDTA pH 8.0) pulsed PIC at a concentration of ∼30 million cells per ml, followed by Branson Sfx150 Sonifier sonication for total ON 4 minutes (1 second ON, 4 seconds OFF; 50% amplitude) to achieve enrichment of 200∼500 bp DNA fragments. Sonicated chromatin was checked by agarose gel electrophoresis or TapeStation 2000 (Agilent) and quantitated with Qubit.

For regular ChIP, 10∼20 μg chromatin and specific antibodies (e.g. SUZ12, JARID2, MTF2, FLAG, H3K27me3, and H2AK119ub1) were incubated overnight with rotation at 4 °C. Following morning, 30 μl protein A/G magnetic beads were added to each ChIP sample, and incubated with rotation at 4 °C for 2 hours. After incubation, the beads were washed 5 times; once with low salt buffer (2 mM EDTA, 20 mM Tris, 1% Triton, 0.1% SDS and 150 mM NaCl), twice with high salt buffer (2 mM EDTA, 20 mM Tris, 1% Triton, 0.1% SDS and 500 mM NaCl) and twice with TE (1 mM EDTA and 10 mM Tris). After the last wash, beads were suspended in 200 μl elution buffer (1% SDS, 0.1 M NaHCO_3_) and incubated for 15 minutes with rotation at room temperature followed by incubation in the thermomixer for 10 minutes with gentle shaking (500 rpm) at 37 °C. The eluted ChIP supernatant was transferred to a new EP tube and incubated in a thermomixer overnight with 1000 rpm shaking at 65°C to remove the crosslinks. Then, RNase A was added and incubated for 1 hour at 37 °C with shaking; followed by Proteinase K treatment for 2 hours at 55 °C with shaking. ChIP DNA was purified using the Qiagen MinElute PCR purification kit and measured with the Qubit High-Sensitive DNA assay kit. Quantitative PCR was performed to check the ChIP efficacy with suitable primers.

In addition, quantitative ChIP-Rx was performed using a modified published approach.^20^^,^^97^ Briefly, 10% human NT2 chromatin was added to mouse chromatin lysate. Alternatively, spike-in chromatin of *Drosophila* S2 cells (Active Motif, 53083) and spike-in antibody H2Av (Active Motif, 61686) were added to mouse chromatin according to the manufacturer’s protocol. Mixed chromatin was treated as single regular ChIP-seq experiment until the completion of DNA sequencing.

#### 4C-seq assay

Circularized chromosome conformation capture (4C) assays were performed using a modified published approach.^98^^,^^99^ Briefly, 10 million detached ESCs or EpiLCs were fixed with 1% final formaldehyde for 10 minutes and quenched with final 0.125 M glycine. The cells were lysed in the 30 ml lysis buffer (50 mM Tris pH 7.5, 150 mM NaCl, 0.5% NP-40, 1% Triton, 5 mM EDTA, and proteinase inhibitors: 2 μg/mL Aprotinin, 1 μg/mL Leupeptin, 1mM PMSF) with rotation at 4 °C for 45 minutes. Isolated nuclei were then fully digested by restricted enzyme DpnII overnight digestion followed by overnight ligation with 50 U T4 DNA ligase at cool room temperature (18∼20 °C). Ligated circular DNA were purified after de-crosslinking incubation with proteinase K at 65 °C for overnight, followed by further incubation with Rnase A at 37 °C for 1 hour. The purified DNA was further digested with restricted enzyme NlaIII and then ligated again and purified. The 4C libraries were amplified with the viewpoint-specific primers with inverse PCR using Roche Expand Long Template PCR System. For each viewpoint, at least 8 PCR reaction products were pooled to enhance the library complexity. The 4C PCR products were purified using Qiagen Quick PCR purification Kit. *Tbx3* promoter viewpoint primers are AAGGGAAGAAGCTGCAGATC (Reading primer) and TGAAGGGAGCCCCACATG.

#### ChIP-seq and 4C-seq library preparation

ChIP-seq and 4C-seq library preparation was performed using the NEBNext Ultra II DNA Library Kit for Illumina (E7645) according to the manufacturer’s protocol. Briefly, 5 ng DNA for ChIP-seq (50 ng DNA for 4C-seq) was incubated with end-repair and A-tailing buffer and enzyme and incubated first for 30 minutes at 20 °C and then for 30 minutes at 65 °C. Then adapters, DNA ligation mix, ligation enhancer were added and incubated at 20 °C for 15 minutes, following USER enzyme treatment at  37 °C for 15 minutes. Post-ligation clean-up was performed using AMPure XP beads. 87 μl (0.9x) beads were added to ligated DNA by gentle pipetting up-down a few times and incubated for 10 minutes at room temperature, then washed with freshly made 80% ethanol twice, eluted in 15 μl 0.1x TE buffer. Library DNA was PCR amplified for 7∼8 cycles (4∼5 cycles for 4C-seq library) with unique index primers and purified with AMPure beads. ChIP-seq libraries were sequenced using Illumina NextSeq 500 platform with 75-bp single-end module.

#### RNA-seq library preparation

RNA-Seq library preparation was performed using the NEBNext Poly(A) mRNA Magnetic Isolation Module (E7490) and NEBNext Ultra RNA Library Prep Kit for Illumina (E7770), according to the manufacturer’s protocol. Prior to starting library preparation, RNA integrity quality was confirmed by RNA ScreenTape with TapeStation (RIN > 9). Total 1 μg RNA was used for library preparation. Following poly(A) RNA enrichment, RNA was fragmented to a size of ∼290 bp, and reverse transcribed to double-strand cDNA. Following cDNA end-repair and A-tailing and adaptor ligation, library DNA was amplified with index primers. Purified library DNA was quantified using the Qubit, and size distributions were measured on a TapeStation (Agilent). RNA-seq library was sequenced with Illumina NextSeq 500 platform.

#### ChIP-seq analysis

ChIP-seq reads quality was confirmed by fastqc,^82^ then high-quality reads were aligned to reference mouse genome (mm10) by bowtie2.^80^ PCR duplicate reads were removed by Picard. For UCSC genome browser visualization, bigwigs were generated by BamCoverage with extending to 200 bp and RPGC normalization.^86^

ChIP-Rx seq reads quality was confirmed by fastqc, then high-quality reads were aligned to target reference mouse genome pulsed spike-in genome (mm10+hg38 or mm10+dm6) by bowtie2 with MAPQ > 2.^97^ The aligned bam files were split to target bam (mm10) and spike-in bam (hg38 or dm6). PCR duplicate reads were removed by Picard for both target and spike-in bam files. The duplicates-removed spike-in bam files mapped reads number was counted and used for generating the normalization factor (a). Generally, normalization factor (a) was calculated by 1 divide the spike-in genome bam file reads count. ChIP-Rx bigwig files were generated by BamCoverage with times normalization factor (a).^86^ For H3K27me3 ChIP-Rx analysis of Tazemetostat treated cells, the normalization factor was normalized to background H3K27me3 regions.^100^ We used the top 2000 H3K27ac peaks as the H3K27me3 background regions for this normalization.

For peak calling, H3K27me3 and H2AK119ub1 peaks were called by macs2 with broad cut-off 0.05; Polycomb peaks were called with narrow cut-off 0.05 setting.^83^ To identify Polycomb target genes, the genes of TSS ±2 kb overlapped with polycomb peaks were regarded as Polycomb target genes. To classify the three groups of Polycomb target genes (see Table S1), the SUZ12 signal of Polycomb targets in ESC and EpiLC (n=2, J2WT and PclWT) was compared using DESeq2 with cut-off (Log_2_FC > 1 and p-value<0.05). The ‘Recruited’ Polycomb target genes were identified using log_2_FC (EpiLC/ESC) > 1; and the ‘Displaced’ Polycomb target genes were identified using Log_2_FC (EpiLC/ESC) < -1. The overlap pie chart analyses of SUZ12, MTF2 and JARID2 ChIP-Rx was plotted using the Intervene tool.^93^

#### CpG islands and Bio-CAP data analysis

CpG islands (CGI) were adapted from ESC Bio-CAP peaks GSE43512.^65^ The Bio-CAP reads were processed according to Long et al. pipeline, which is similar to ChIP-seq. Briefly, the reads were aligned to the mouse genome (mm10) with bowtie2. PCR duplicates were removed using Picard MarkDuplicates. The bigwig file was generated by BamCoverage with RPGC normalization. To investigate the Polycomb binding around CGI in MTF2-lack ESC, or EpiLC, the distance between Polycomb ChIP peak center and CGI was determined by bedtools intersect. The distances in relevant ESC and EpiLC were plotted as boxplot. The merged Polycomb peaks were extended from the center to 5 kb up- and down-streaming with total length of 10 kb. Because the Polycomb protein peaks are narrow and H2AK119ub1 peaks are broad, to investigate the correlation among ChIP-seq samples, the 2 kb-bins were introduced to calculate the correlation. Then extended peaks were spitted to 5 equal bins (2 kb bin), the mapped reads count of each 2-kb bin was measured by multiBamSummary with the option of -bed. The correlation heatmap of ChIP-seq samples was plotted with R pheatmap.

#### RNA-seq analysis

RNA-seq reads quality was confirmed by fastqc, then high quality reads aligned to reference mouse transcriptome (Ensembl GRCm38.101) with STAR2.7.1.^87^ The transcriptome aggregated reads counts were combined a metatable for down-streaming analysis. Differentially expressed genes (DE genes) were identified using DESeq2.^88^ Removed the low-expressed genes (BaseMean < 15), the DE genes were counted with cut-off 2 folds change (Log_2_FC> 1 or Log_2_FC <-1) and padj<0.05. For visualization on the UCSC Genome Browser, bigwigs were generated by BamCoverage with no extend and RPKM normalization.^86^

#### 4C-seq analysis

4C-seq fastq was trimmed to same length and confirmed the quality using fastqc. 4C-seq data was analysied using the full analysis pipeline pipe4C.^92^ Briefly, the index information text file was edited according to the restrict enzyme and specific bait information. pipe4C will process data, identify and trim read primer fastq, then align to mouse genome mm10. The analysis module was *cis*, which analyze the site 2 Mb region around the bait, provide normalized bigwig and wig files. The quantitative 4C-seq signal at H3K27ac and SUZ12 sites was measured from normalized 4C bigwig files.

### Quantification and statistical analysis

Data obtained from RT-qPCR and RNA-seq quantifications were analyzed using a two-tailed Student’s t-test assuming unequal variances. Correlations were analyzed using the Pearson’s method. Numbers of experimental replicates, P-values and the tests can be found in the figure legends.

## Data Availability

• Newly generated high-throughput sequencing data have been deposited at GEO and are publicly available as of the date of publication. Accession numbers are listed in the key resources table. Original western blot and gel images reported in this paper have been deposited at Mendeley and are publicly available as of the date of publication. The DOI is listed in the key resources table. • No new software was developed during this study. • Any additional information required to reanalyze the data reported in this paper is available from the lead contact upon request.

## Supplemental information

Document S1. Figures S1–S7 and supplemental references

Table S1. Three group of PcG targets, related to Figure 1

Document S2. Article plus supplemental information
