# Supplementary material for: PRC2.1- and PRC2.2-specific accessory proteins drive recruitment of different forms of canonical PRC1
Source: Mol Cell. 2023 May 4;83(9):1393–1411.e7. doi: 10.1016/j.molcel.2023.03.018 (PMC10168607; doi:10.1016/j.molcel.2023.03.018)
Supplement: Document S1. Figures S1–S7 and supplemental references [file mmc1.pdf]

Molecular Cell, Volume *83*

Supplemental information

PRC2.1- and PRC2.2-specific accessory proteins drive recruitment of different forms of canonical PRC1

Eleanor Glancy, Cheng Wang, Ellen Tuck, Evan Healy, Simona Amato, Hannah K. Neikes, Andrea Mariani, Marlena Mucha, Michiel Vermeulen, Diego Pasini, and Adrian P. Bracken

Supplemental information

PRC2.1 and PRC2.2 Specific Accessory Proteins Drive Recruitment of Different Forms of Canonical PRC1.

Eleanor Glancy, Cheng Wang, Ellen Tuck, Evan Healy, Simona Amato, Hannah K. Neikes, Andrea Mariani, Marlena Mucha, Michiel Vermeulen, Diego Pasini, Adrian P. Bracken.

Figure S1. Polycombs and histone modifications during ESC to EpiLC differentiation, Related to Figure 1.

(A) Western blot analyses on nuclear lysates from ESCs and EpiLCs, using the indicated antibodies.

(B) Bar plot representing mRNA abundance of genes encoding PRC2 and PRC1 subunits in WT ESCs (blue) and EpiLCs (orange). Error bars show standard deviation (n=3).

(C) Pie charts showing the overlap fraction of 'displaced', 'maintained', and 'recruited' sites from JARID2, MTF2 and SUZ12 ChIP-Rx analyses, respectively. The colour and size of pie indicate the fraction of overlap.

(D) Heatmaps of H3K27me3, H2AK119ub1, CBX7 and H3K27ac ChIP-Rx enrichments with normalized read densities from three categories of SUZ12 target genes, identified in Figure 1B.

Figure S1.

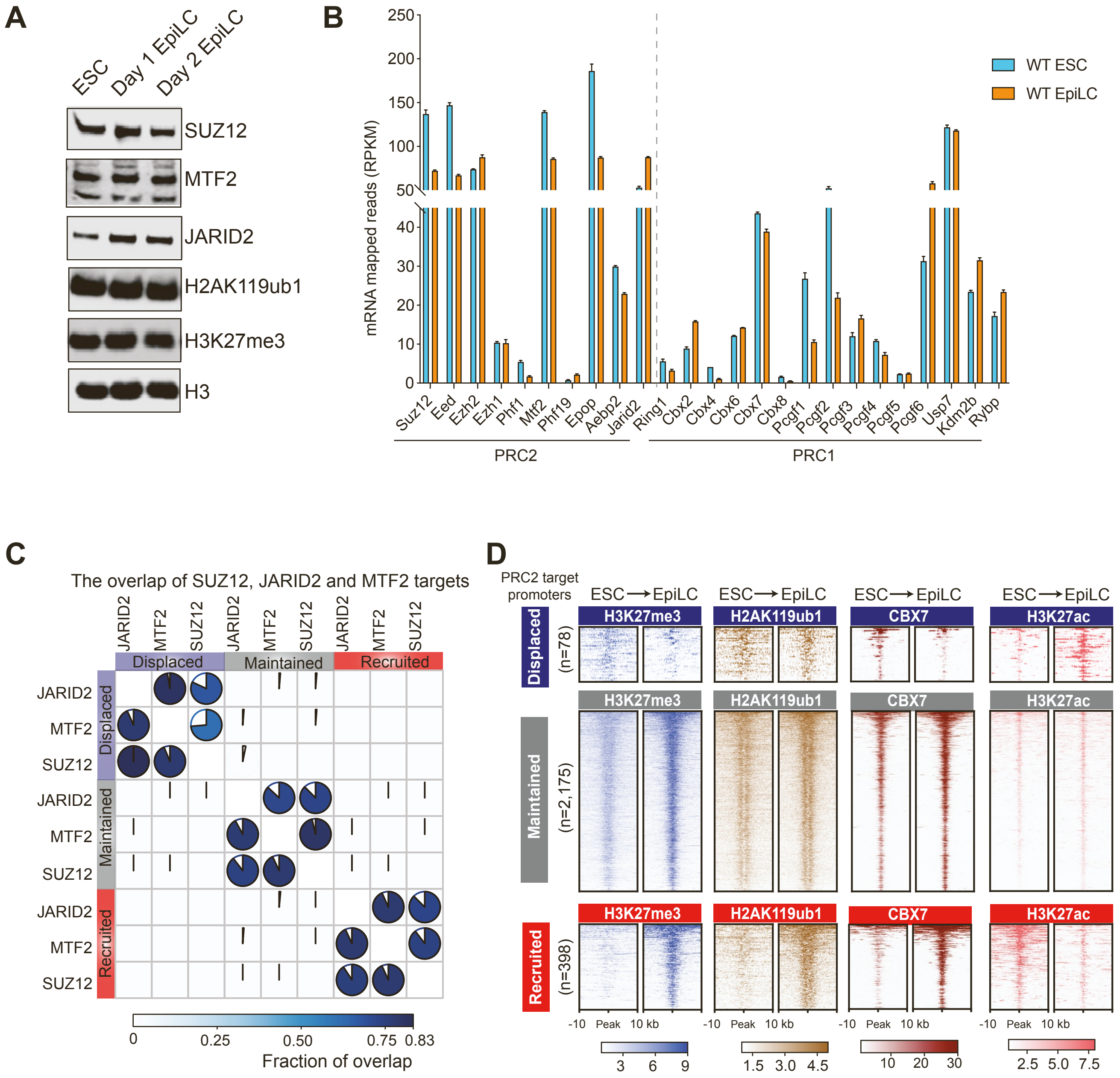

A
ESC
Day 1 EpiLC
Day 2 EpiLC
SUZ12
MTF2
JARID2
H2AK119ub1
H3K27me3
H3
B
mRNA mapped reads (RPKM)
WT ESC
WT EpiLC
Suz12 Eed Ezh2 Ezh1 Phf1 Mtf2 Phf19 Epop Aebp2 Jarid2 Ring1 Cbx2 Cbx4 Cbx6 Cbx7 Cbx8 Pcgf1 Pcgf2 Pcgf3 Pcgf4 Pcgf5 Pcgf6 Usp7 Kdm2b Rybp
PRC2
PRC1
C
The overlap of SUZ12, JARID2 and MTF2 targets
Displaced
Maintained
Recruited
JARID2
MTF2
SUZ12
Fraction of overlap
0 0.25 0.50 0.75 0.83
D
PRC2 target promoters
ESC → EpiLC
H3K27me3
H2AK119ub1
CBX7
H3K27ac
Displaced (n=78)
Maintained (n=2,175)
Recruited (n=398)
-10 Peak 10 kb
3 6 9
1.5 3.0 4.5
10 20 30
2.5 5.0 7.5

Figure S2. PRC2.1 drives H3K27me3 deposition while PRC2.2 drives CBX7-cPRC1 recruitment to Polycomb target genes, Related to Figure 2.

(A) Genome browser tracks showing ChIP-Rx profiles of the indicated antibodies in WT ESCs; WT, J2KO, TKO and QKO EpiLCs at the 'Recruited' *Pdgfa* gene locus. (B) Average and tornado plots showing ChIP-Rx and ChIP-seq enrichments of the indicated antibodies at 'Maintained' Polycomb target genes (n=2,175) in WT, J2KO, TKO and QKO EpiLCs.

(C) Tornado plots showing CBX7 ChIP-Rx enrichments on 'Maintained' Polycomb target genes in WT and J2KO EpiLCs, generated from a different ESC strain (E14).

(D) As in C for WT, J2KO, TKO and QKO ESCs.

(E) Tornado plots showing CBX7 ChIP-Rx enrichments generated using an alternative antibody (Millipore) on 'Maintained' Polycomb target genes in WT, J2KO, TKO and QKO EpiLCs (n=2,175).

(F) Tornado plots showing JARID2 ChIP-Rx enrichments on 'Maintained' Polycomb target genes in WT and *Pcgf2/4* KO EpiLCs.

(G) Bar plot representing the mRNA abundance of *Cbx7* (RPKM from RNA-seq) in relevant cell lines. Error bars represent standard deviation (n=3)

(H) Western blot analysis of total protein extracts from WT, J2KO, TKO and QKO ESCs, using the indicated antibodies.

Figure S2.

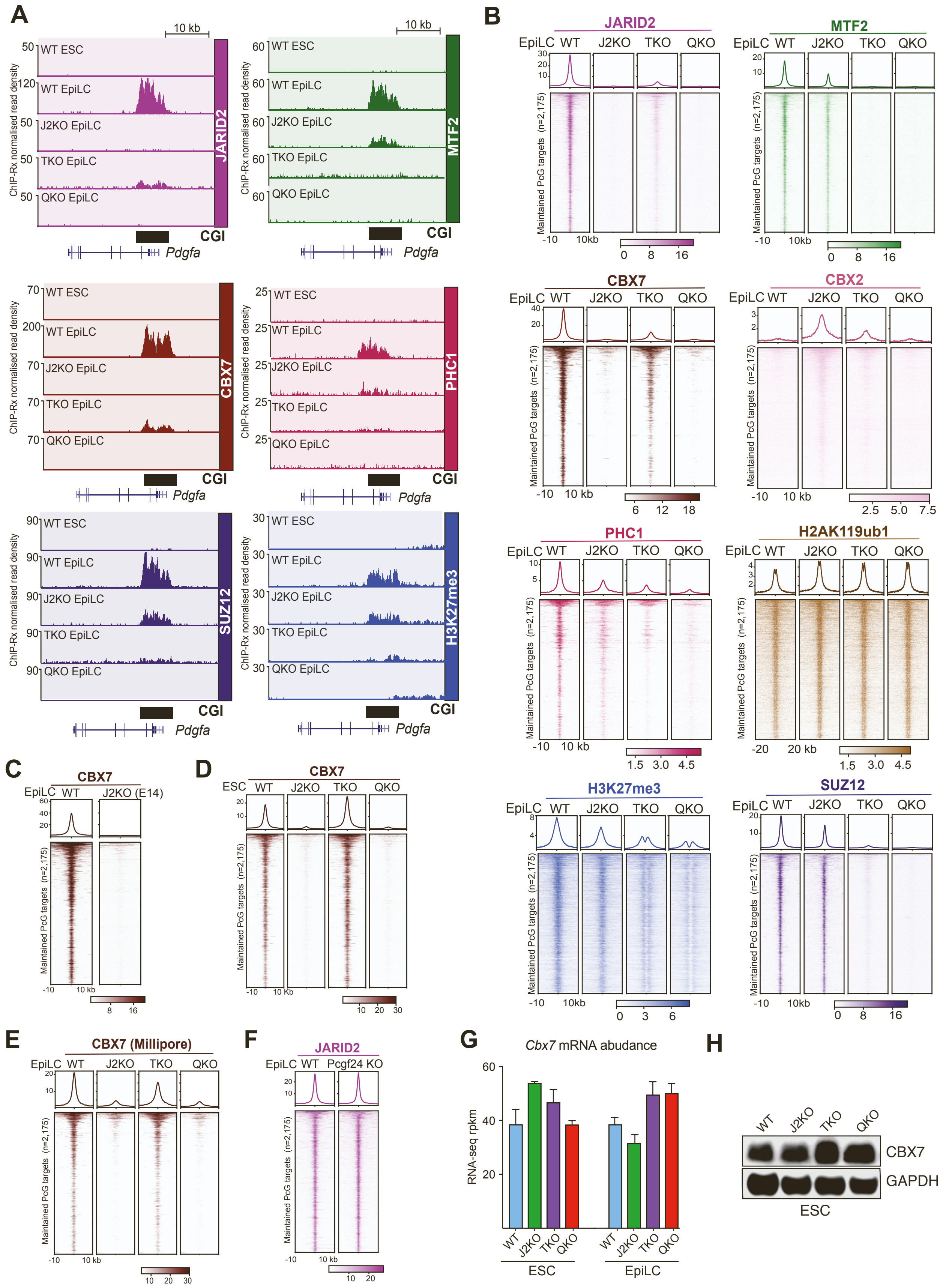
A
10 kb
WT ESC
WT EpiLC
J2KO EpiLC
TKO EpiLC
QKO EpiLC
ChIP-Rx normalised read density
JARID2
MTF2
CBX7
PHC1
SUZ12
H3K27me3
CGI
Pdgfa
B
JARID2
MTF2
CBX7
CBX2
PHC1
H2AK119ub1
H3K27me3
SUZ12
EpiLC WT J2KO TKO QKO
Maintained PcG targets (n=2,175)
-10 10kb
C
CBX7
EpiLC WT J2KO (E14)
D
CBX7
ESC WT J2KO TKO QKO
E
CBX7 (Millipore)
EpiLC WT J2KO TKO QKO
F
JARID2
EpiLC WT Pcgf24 KO
G
Cbx7 mRNA abudance
RNA-seq rpkm
WT J2KO TKO QKO
ESC
EpiLC
H
WT J2KO TKO QKO
CBX7
GAPDH
ESC

Figure S3. Rescue of PRC2.1 or PRC2.2 recruitment with ectopic expression of MTF2 or JARID2, respectively, Related to Figure 3.

(A) Western blot analyses using the indicated antibodies on FLAG immunoprecipitations and input lysates from QKO ESCs, and from QKO ESCs either ectopically expressing FLAG-MTF2 or FLAG-JARID2.

(B) ChIP-qPCR analysis of AEBP2, EPOP and IgG in WT, QKO, QKO + FLAG-MTF2 and QKO + FLAG-JARID2 ESCs. Error bars represent standard deviation of technical triplicates.

(C) Average plots of ChIP-Rx enrichments for JARID2, CBX7, SUZ12 and MTF2 and ChIP-seq enrichments of PHC1 in QKO, and QKO ESCs ectopically expressing either FLAG-MTF2 or FLAG-JARID2 at PRC2.1 only regions (top) and PRC2.1/PRC2.2 shared regions (bottom). The PRC2.1/PRC2.2 shared and PRC2.1 only regions were taken from Healy *et al.*, 2019[1].

(D) Box plot of PHC1 ChIP-seq signal in QKO and QKO ESCs ectopically expressing either FLAG-MTF2 or FLAG-JARID2 at PRC2.1/PRC2.2 shared regions. The *** indicates a p-value < 0.001.

(E) ChIP-qPCR analysis of CBX7 and JARID2 in WT, QKO, QKO + JARID2 and QKO + JARID2-K116R ESCs. Error bars represent standard deviation of technical triplicates.

(F) Western blot analyses using the indicated antibodies on whole cell lysates from WT and *Aebp2*$^{GT/GT}$ ESCs.

(G) ChIP-qPCR analysis of CBX7 in WT and *Aebp2*$^{GT/GT}$ ESCs. Error bars represent standard deviation of technical triplicates.

(H) Endogenous IP-MS using CBX7 as bait. Table of iBAQ values (units x10^7) of PRC1 and PRC2-associated proteins identified in endogenous co-IPs of CBX7 performed on E14 mouse ESCs (n=3).

Figure S3.

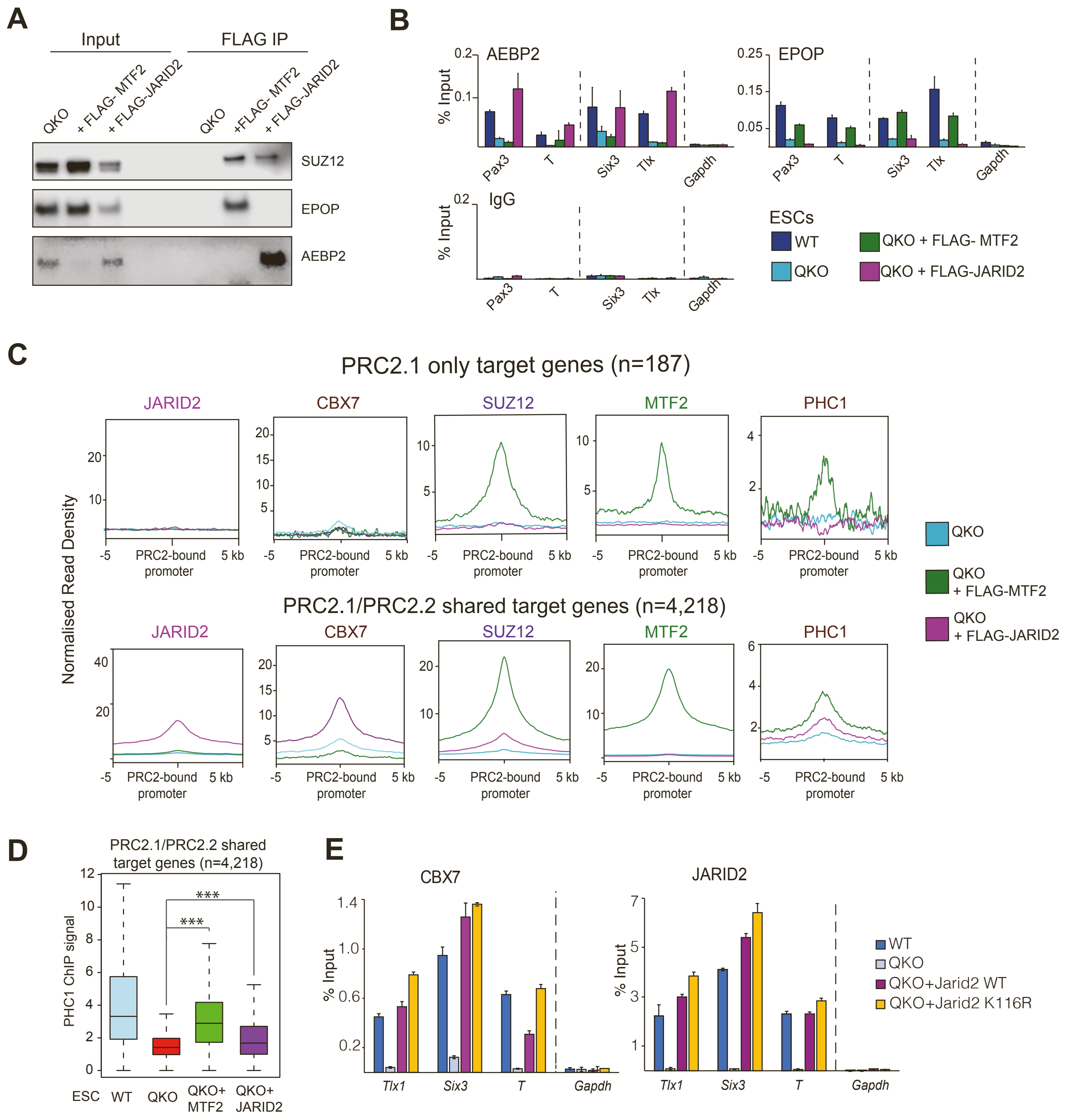

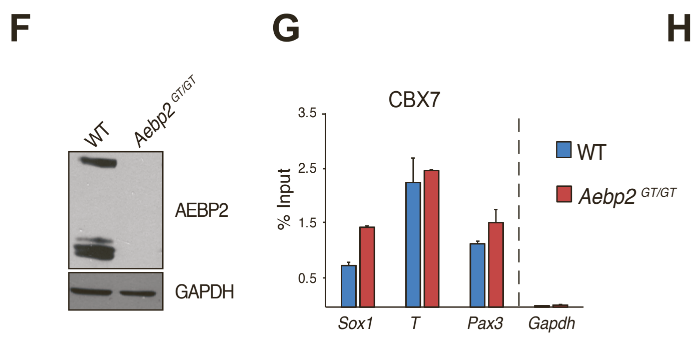

H

		CBX7 IP-MS			IgG IP-MS		
		Rep1	Rep2	Rep3	Rep1	Rep2	Rep3
cPRC1	Cbx7	293.0	235.0	255.5	0.0	0.0	0.0
	Phc1	347.9	258.6	341.1	0.1	0.3	0.3
	Pcgf2	160.5	114.2	196.2	0.0	0.0	0.1
	Pcgf4	51.5	41.7	67.7	0.0	0.0	0.0
	Phc2	15.5	20.5	12.7	0.0	0.0	0.0
	Phc3	2.7	2.3	1.6	0.0	0.0	0.0
PRC1	Ring1b	514.3	486.9	562.6	1.0	1.7	1.3
	Ring1	0.7	0.3	0.6	0.0	0.0	0.1
vPRC1	Pcgf6	13.1	8.0	11.0	0.6	0.6	0.9
	Pcgf1	2.8	2.4	2.5	0.2	0.5	0.3
	Rybp	0.2	0.5	0.4	0.2	0.8	0.4
PRC2	Eed	2.2	2.1	2.7	1.4	4.9	1.2
	Ezh2	1.2	0.7	1.5	0.6	1.7	0.9
	Suz12	1.8	0.8	1.2	0.6	1.9	1.3
	Mtf2	1.1	0.6	1.1	0.3	1.4	0.5
	Jarid2	0.2	0.2	0.2	0.0	0.3	0.1

Figure S4. Consequences of pharmacological or genetic mediated depletion of H3K27me3, Related to Figure 4.

(A) Average and tornado plots showing ChIP-Rx enrichments of SUZ12, MTF2 and H2AK119ub1 at 'Maintained' Polycomb targets (n=2,175) in ESCs treated for 7 days with Tazemetostat (TAZ) or DMSO control.

(B) Genome browser tracks of representative genes from Quintile 1 (*Nat8l*) and Quintile 5 (*Spata3*), showing ChIP-Rx of SUZ12, MTF2 and H2AK119ub1 in ESCs treated for 7 days with Tazemetostat or DMSO control.

(C) Genome browser tracks of representative genes from Quintile 1 (*Frem2 and Cnr1*) and Quintile 5 (*Sgpp2 and Dbx2*), showing ChIP-Rx of CBX7, JARID2, H3K27me3, SUZ12, MTF2 and H2AK119ub1 in ESCs treated for 7 days with Tazemetostat or DMSO control.

(D) Average and tornado plots showing ChIP-seq enrichments of SUZ12 in WT, *Ezh1/2* dKO and *Ezh1*-KO / *Ezh2-Y726D* ESCs at 'maintained' Polycomb targets (n=2,175) grouped into quintiles based on CBX7 abundance difference between DMSO control or Tazemetostat treated cells, as described in Figure 4C.

(E) Genome browser tracks of representative genes from Quintile 1 (*Nat8l*) and Quintile 5 (*Spata3*), showing ChIP-seq enrichments of SUZ12 in WT, *Ezh1/2* dKO and *Ezh1*-KO / *Ezh2-Y726D* ESCs.

Figure S4.

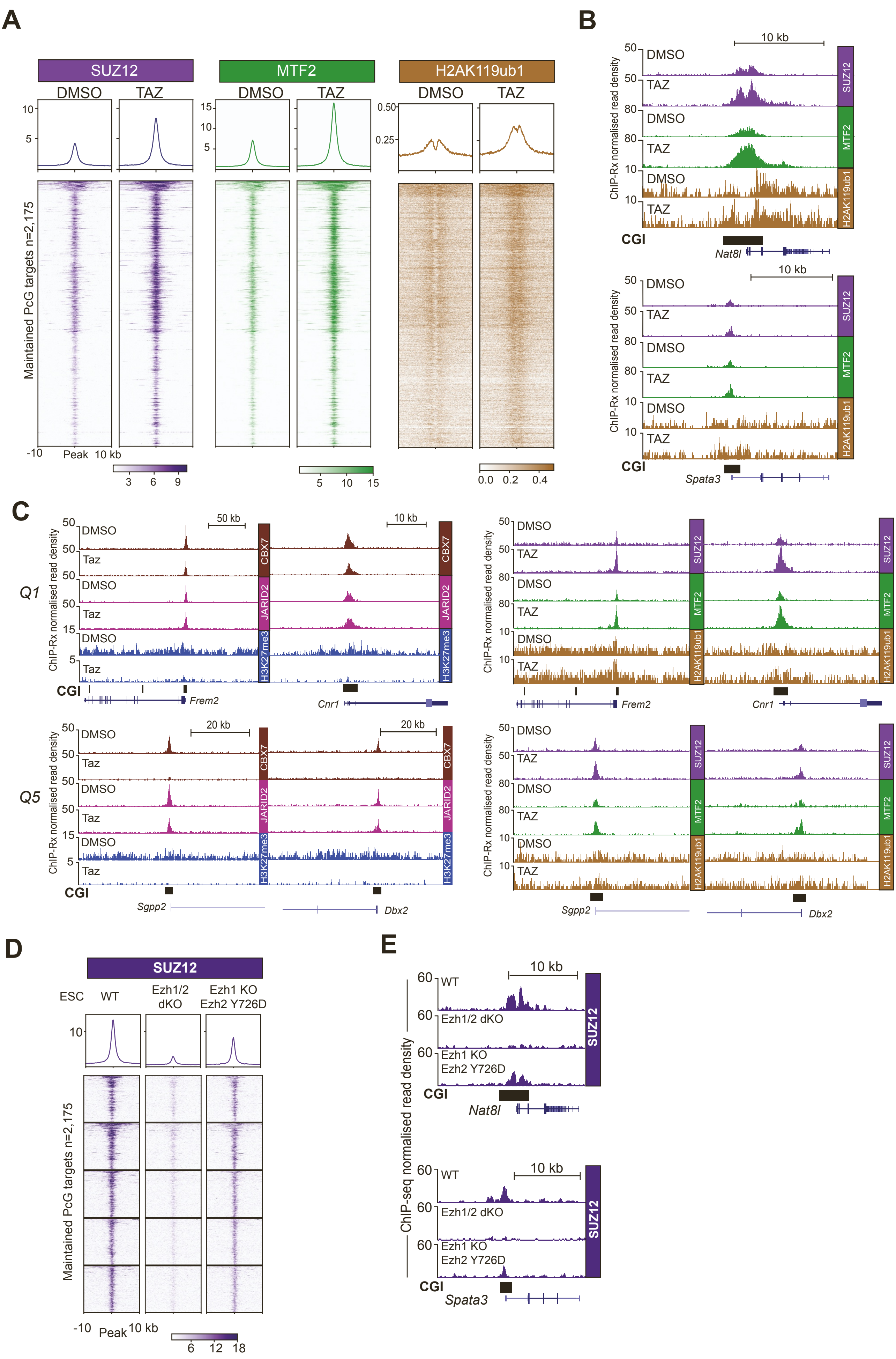

A
SUZ12
MTF2
H2AK119ub1
DMSO
TAZ
Maintained PcG targets n=2,175
-10
Peak
10 kb
B
10 kb
DMSO
TAZ
ChIP-Rx normalised read density
CGI
Nat8l
Spata3
C
Q1
Q5
CBX7
JARID2
H3K27me3
Frem2
Cnr1
Sgpp2
Dbx2
50 kb
20 kb
D
ESC
WT
Ezh1/2 dKO
Ezh1 KO Ezh2 Y726D
E
ChIP-seq normalised read density

Figure S5. Contrasting PRC2.1 and PRC2.2 binding profiles consistent with independent recruitment mechanisms, Related to Figure 5.

(A) Genome browser tracks showing ChIP-Rx binding profiles of JARID2 in WT ESCs, WT, J2KO, TKO and QKO EpiLCs, and WT EpiLC H2AK119ub1 at the extended *HoxC* locus.

(B) Genome browser tracks showing ChIP-Rx binding profiles of MTF2 in WT ESCs, WT, J2KO, TKO and QKO EpiLCs, and ESC Bio-CAP (Long *et al.*, 2013[2]) at the extended *HoxC* locus.

(C) Genome browser tracks showing ChIP-Rx binding profiles of SUZ12 generated in this manuscript (purple), compared to those generated from Hojfeldt *et al.*, 2019[3] (blue) and Healy *et al.*, 2019[1] (dark cyan). Bio-Cap profiles (pink) and H2AK119ub1 (gold) are also included.

(D) Genome browser tracks showing SUZ12 ChIP-Rx enrichment in WT, TKO and J2KO EpiLCs as well as a merged track for TKO + J2KO SUZ12 enrichment, aligned with H2AK119ub1 ChIP-Rx and Bio-CAP CGI enrichment in WT EpiLCs at the expanded *HoxC* cluster.

(E) Heatmap representing the Pearson correlations between each of the indicated ChIP-Rx analyses in WT, J2KO and TKO EpiLCs, and ESC Bio-CAP (Long *et al.*, 2013[2]).

(F) A model representing the binding of PRC2 in WT, J2KO and TKO EpiLCs. PRC2.1 binding in J2KO cells (lacking PRC2.2) forms ‘sharp’ peak-like profiles, whereas PRC2.2 binding in TKO cells (lacking PRC2.1) forms ‘broad’ profiles.

Figure S5.

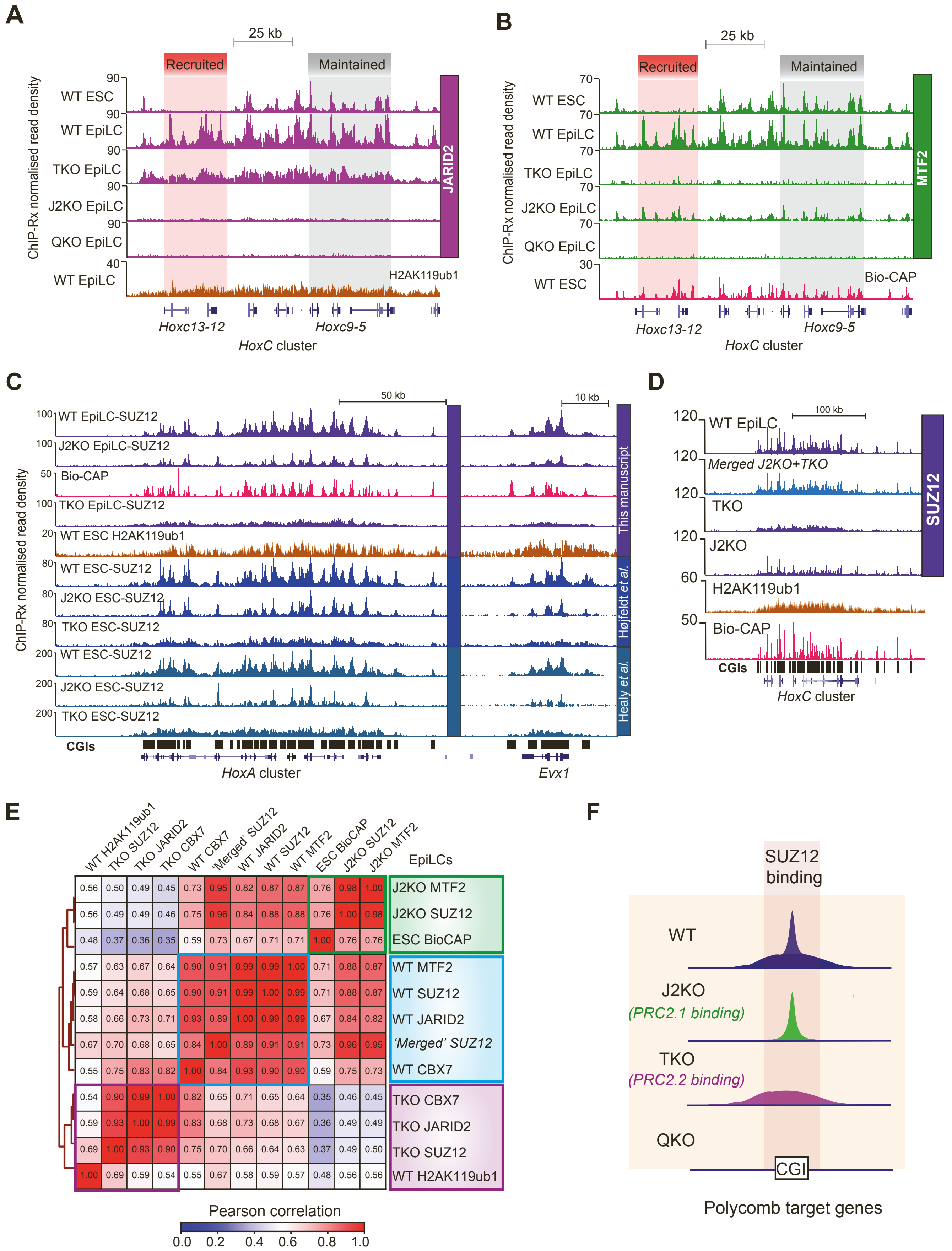
A
25 kb
Recruited
Maintained
ChIP-Rx normalised read density
90
WT ESC
WT EpiLC
TKO EpiLC
J2KO EpiLC
QKO EpiLC
40
WT EpiLC
H2AK119ub1
JARID2
Hoxc13-12
Hoxc9-5
HoxC cluster
B
25 kb
Recruited
Maintained
ChIP-Rx normalised read density
70
WT ESC
WT EpiLC
TKO EpiLC
J2KO EpiLC
QKO EpiLC
30
WT ESC
Bio-CAP
MTF2
Hoxc13-12
Hoxc9-5
HoxC cluster
C
50 kb
10 kb
ChIP-Rx normalised read density
100 WT EpiLC-SUZ12
100 J2KO EpiLC-SUZ12
50 Bio-CAP
100 TKO EpiLC-SUZ12
20 WT ESC H2AK119ub1
80 WT ESC-SUZ12
80 J2KO ESC-SUZ12
80 TKO ESC-SUZ12
200 WT ESC-SUZ12
200 J2KO ESC-SUZ12
200 TKO ESC-SUZ12
This manuscript
Højfeldt et al.
Healy et al.
CGIs
HoxA cluster
Evx1
D
100 kb
120 WT EpiLC
120 Merged J2KO+TKO
120 TKO
120 J2KO
60 H2AK119ub1
50 Bio-CAP
CGIs
HoxC cluster
SUZ12
E
WT H2AK119ub1
TKO SUZ12
TKO JARID2
TKO CBX7
WT CBX7
'Merged' SUZ12
WT JARID2
WT SUZ12
WT MTF2
ESC BioCAP
J2KO SUZ12
J2KO MTF2
EpiLCs
0.56 0.50 0.49 0.45 0.73 0.95 0.82 0.87 0.87 0.76 0.98 1.00 J2KO MTF2
0.56 0.49 0.49 0.46 0.75 0.96 0.84 0.88 0.88 0.76 1.00 0.98 J2KO SUZ12
0.48 0.37 0.36 0.35 0.59 0.73 0.67 0.71 0.71 1.00 0.76 0.76 ESC BioCAP
0.57 0.63 0.67 0.64 0.90 0.91 0.99 0.99 1.00 0.71 0.88 0.87 WT MTF2
0.59 0.64 0.68 0.65 0.90 0.91 0.99 1.00 0.99 0.71 0.88 0.87 WT SUZ12
0.58 0.66 0.73 0.71 0.93 0.89 1.00 0.99 0.99 0.67 0.84 0.82 WT JARID2
0.67 0.70 0.68 0.65 0.84 1.00 0.89 0.91 0.91 0.73 0.96 0.95 'Merged' SUZ12
0.55 0.75 0.83 0.82 1.00 0.84 0.93 0.90 0.90 0.59 0.75 0.73 WT CBX7
0.54 0.90 0.99 1.00 0.82 0.65 0.71 0.65 0.64 0.35 0.46 0.45 TKO CBX7
0.59 0.93 1.00 0.99 0.83 0.68 0.73 0.68 0.67 0.36 0.49 0.49 TKO JARID2
0.69 1.00 0.93 0.90 0.75 0.70 0.66 0.64 0.63 0.37 0.49 0.50 TKO SUZ12
1.00 0.69 0.59 0.54 0.55 0.67 0.58 0.59 0.57 0.48 0.56 0.56 WT H2AK119ub1
Pearson correlation
0.0 0.2 0.4 0.6 0.8 1.0
F
SUZ12 binding
WT
J2KO
(PRC2.1 binding)
TKO
(PRC2.2 binding)
QKO
CGI
Polycomb target genes

Figure S6. DNA and histone modification binding activities of MTF2 and JARID2 facilitate the respective chromatin binding of PRC2.1 and PRC2.2, Related to Figure 6.

(A) Schematic of the amino acid sequences of the EH domains from PHF1, MTF2 and PHF19 of human aligned with the same region of *Drosophila Pcl*.

(B) ChIP-qPCR of the indicated antibodies in parental QKO ESC, as well as QKO ESCs expressing FLAG-MTF2-L, FLAG-MTF2-L-EH^{mut}, FLAG-MTF2-S or FLAG-MTF2-S-EH^{mut}.

(C) Average and tornado plots showing ChIP-Rx enrichments of SUZ12 in the indicated ESC lines at all PRC2 bound promoters.

(D) Boxplot analyses of SUZ12 ChIP-Rx read counts for wild-type (WT), QKO and QKO ESCs rescued with either FLAG-MTF2-L or FLAG-MTF2-S at non-PRC2 associated promoters (n=15,369) and at intergenic regions (n=22,050).

(E) Average and tornado plots showing ChIP-Rx enrichments of SUZ12 in WT, QKO, QKO + JARID2 and QKO + JARID2-ΔUIM ESCs.

(F) Line plots representing the ChIP-Rx enrichment of SUZ12 in WT, QKO, QKO+JARID2 and QKO+JARID2-ΔUIM ESCs relative to their respective levels in WT ESCs.

(G) Genome browser tracks of CBX7 ChIP-Rx in QKO, QKO + JARID2 or QKO + JARID2-ΔUIM at UIM-dependent (left) and UIM-independent (right) gene loci.

(H) Average plots showing ChIP-Rx normalised enrichments for H2AK119ub1, H3K27me3, MTF2 and JARID2 in wild-type ESCs (data from Healy *et al*. 2019[1]) at UIM-dependent (red) and UIM-independent regions, as defined in Figure 6I.

Figure S6.

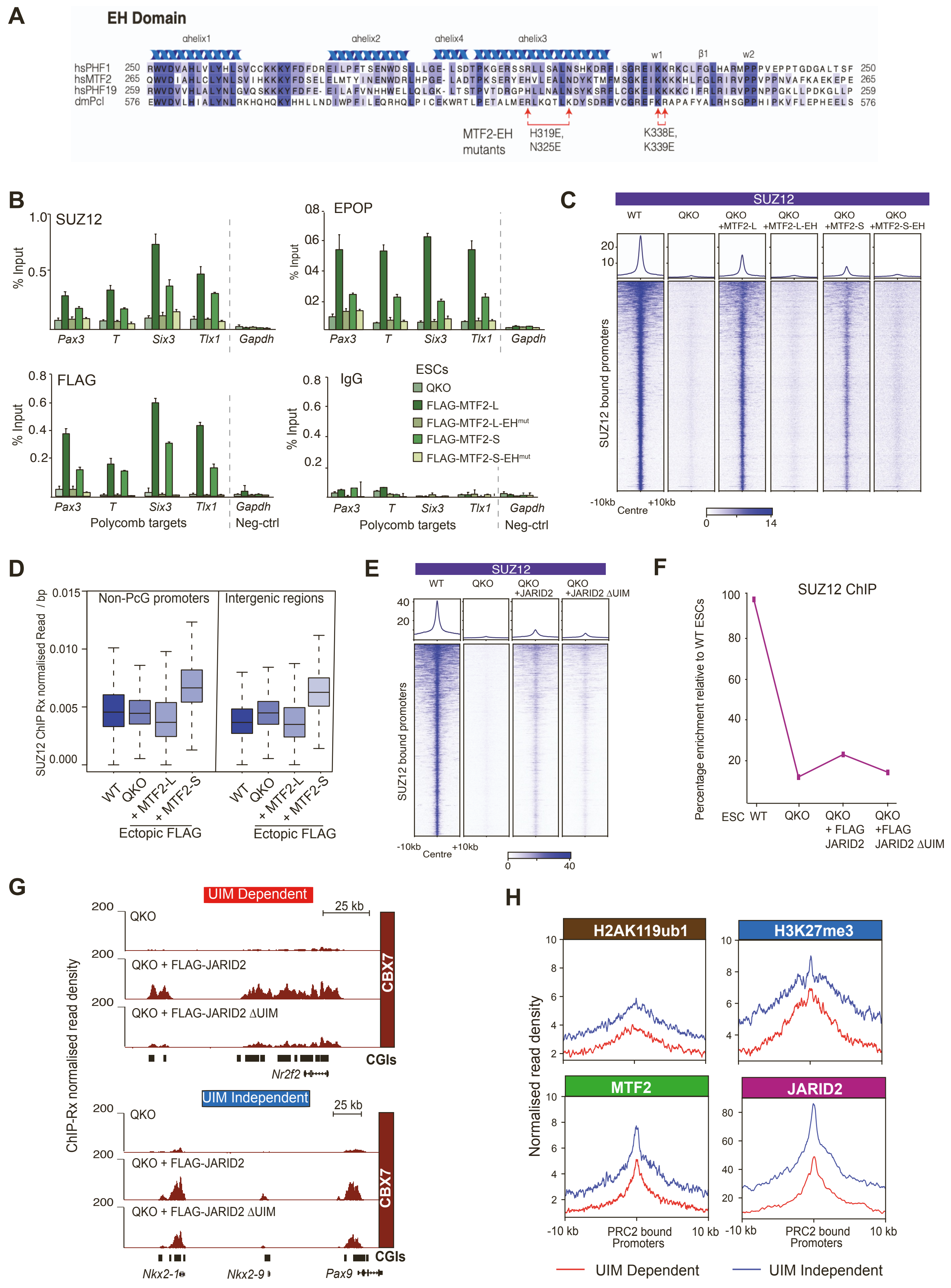

A
EH Domain
αhelix1
αhelix2
αhelix4
αhelix3
w1
β1
w2
hsPHF1 250
hsMTF2 265
hsPHF19 259
dmPcl 576
MTF2-EH mutants
H319E, N325E
K338E, K339E
B
SUZ12
EPOP
FLAG
IgG
% Input
Pax3
T
Six3
Tlx1
Gapdh
Polycomb targets
Neg-ctrl
ESCs
QKO
FLAG-MTF2-L
FLAG-MTF2-L-EHmut
FLAG-MTF2-S
FLAG-MTF2-S-EHmut
C
SUZ12
WT
QKO
QKO +MTF2-L
QKO +MTF2-L-EH
QKO +MTF2-S
QKO +MTF2-S-EH
SUZ12 bound promoters
-10kb
Centre
+10kb
D
SUZ12 ChIP Rx normalised Read / bp
Non-PcG promoters
Intergenic regions
WT
QKO
+ MTF2-L
+ MTF2-S
Ectopic FLAG
E
SUZ12
WT
QKO
QKO +JARID2
QKO +JARID2 ΔUIM
SUZ12 bound promoters
-10kb
Centre
+10kb
F
SUZ12 ChIP
Percentage enrichment relative to WT ESCs
ESC WT
QKO
QKO + FLAG JARID2
QKO +FLAG JARID2 ΔUIM
G
UIM Dependent
UIM Independent
25 kb
ChIP-Rx normalised read density
QKO
QKO + FLAG-JARID2
QKO + FLAG-JARID2 ΔUIM
CBX7
CGIs
Nr2f2
Nkx2-1
Nkx2-9
Pax9
H
H2AK119ub1
H3K27me3
MTF2
JARID2
Normalised read density
-10 kb
PRC2 bound Promoters
10 kb
UIM Dependent
UIM Independent

Figure S7. PRC2.1 and PRC2.2 contributions on the extended *Tbx3* gene locus and on Polycomb target repression, Related to Figure 7.

(A) Genome browser tracks showing H3K27ac ChIP-seq profile in WT ESCs, and WT, J2KO, TKO and QKO EpiLCs. Also shown are P300 and OCT4 ChIP-seq profiles in WT ESCs and EpiLCs at the extended *Tbx3* gene locus around the E1, E2, and E3 sites. The P300 and OCT4 ChIP-seq data were downloaded from Buecker *et al.*, 2014[4].

(B) Genome browser tracks showing ChIP-Rx profiles of JARID2 and MTF2 in WT ESCs, WT, J2KO, TKO and QKO and QKO EpiLCs at the extended *Tbx3* gene locus around the E1, E2, and E3 sites.

(C) Bar plots representing the mRNA levels (RPKM from RNA-seq) of 'Recruited' Polycomb target gene *Tbx3*, as well as the 'Maintained' target genes *Tbx5* and *Lhx5* in WT ESCs, WT EpiLCs and QKO EpiLCs. Error bars represent standard deviation (n=3).

(D) Boxplots representing ChIP-seq signal of H3K27Ac at 'recruited' targets (n=398) in WT ESCs, WT, J2KO, TKO, QKO and *Pcgf2/4* KO EpiLCs. The *** represents a p-value < 0.001.

(E) ChIP-qPCRs of CBX7 at the S1, S2 and S3 regions, as well as the*Tbx3* promoter and negative control *Gapdh* promoter in WT and *Pcgf2/4* KO EpiLCs. Error bars represent standard deviation of technical triplicates.

(F) Volcano plots from RNA-seq comparisons of J2KO vs matched WT EpiLCs (left; called 'no PRC2.2'), TKO vs matched WT EpiLCs (middle; called 'no PRC2.1') and QKO vs matched WT EpiLCs (right; called 'no PRC2.1 & PRC2.2'). Indicated inside the panel are the numbers of differentially expressed (DE) genes, with a cut-off (lLog2FCl>1, padj<0.05 by DESeq2). The number of Polycomb (PcG) target genes that are "Up" or "Down" is also indicated. (n=3).

Figure S7.

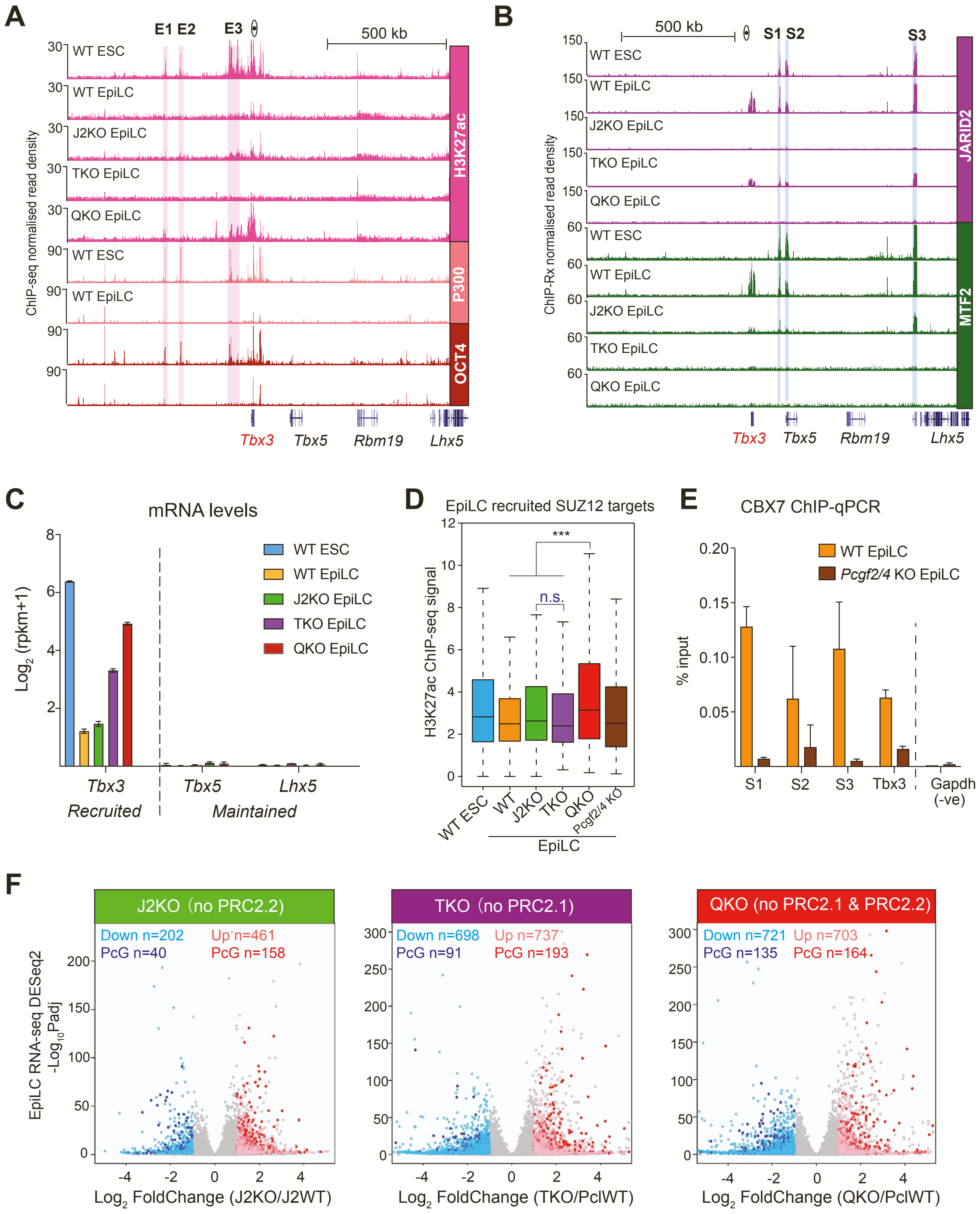
A
E1 E2
E3
500 kb
WT ESC
WT EpiLC
J2KO EpiLC
TKO EpiLC
QKO EpiLC
H3K27ac
P300
OCT4
ChIP-seq normalised read density
Tbx3
Tbx5
Rbm19
Lhx5
B
500 kb
S1 S2
S3
JARID2
MTF2
ChIP-Rx normalised read density
C
mRNA levels
Log2 (rpkm+1)
WT ESC
WT EpiLC
J2KO EpiLC
TKO EpiLC
QKO EpiLC
Recruited
Maintained
D
EpiLC recruited SUZ12 targets
H3K27ac ChIP-seq signal
***
n.s.
WT ESC
WT
J2KO
TKO
QKO
Pcgf2/4 KO
EpiLC
E
CBX7 ChIP-qPCR
% input
WT EpiLC
Pcgf2/4 KO EpiLC
S1
S2
S3
Tbx3
Gapdh (-ve)
F
J2KO (no PRC2.2)
Down n=202
PcG n=40
Up n=461
PcG n=158
Log2 FoldChange (J2KO/J2WT)
TKO (no PRC2.1)
Down n=698
PcG n=91
Up n=737
PcG n=193
Log2 FoldChange (TKO/PclWT)
QKO (no PRC2.1 & PRC2.2)
Down n=721
PcG n=135
Up n=703
PcG n=164
Log2 FoldChange (QKO/PclWT)
EpiLC RNA-seq DESeq2 -Log10Padj

References.

1. Healy, E., Mucha, M., Glancy, E., Fitzpatrick, D.J., Conway, E., Neikes, H.K., Monger, C., Van Mierlo, G., Baltissen, M.P., Koseki, Y., Bracken, A.P. (2019). PRC2.1 and PRC2.2 Synergize to Coordinate H3K27 Trimethylation. Molecular Cell 70, 437-452. 10.1016/j.molcel.2019.08.012.

2. Long, H.K., Sims, D., Heger, A., Blackledge, N.P., Kutter, C., Wright, M.L., Grutzner, F., Odom, D.T., Patient, R., Ponting, C.P., and Klose, R.J. (2013). Epigenetic conservation at gene regulatory elements revealed by non-methylated DNA profiling in seven vertebrates. eLife 2, e00348. 10.7554/eLife.00348.

3. Hojfeldt, J.W., Hedehus, L., Laugesen, A., Tatar, T., Wiehle, L., and Helin, K. (2019). Non-core Subunits of the PRC2 Complex Are Collectively Required for Its Target-Site Specificity. Molecular cell. 10.1016/j.molcel.2019.07.031.

4. Buecker, C., Srinivasan, R., Wu, Z., Calo, E., Acampora, D., Faial, T., Simeone, A., Tan, M., Swigut, T., and Wysocka, J. (2014). Reorganization of enhancer patterns in transition from naive to primed pluripotency. Cell Stem Cell 14, 838-853. 10.1016/j.stem.2014.04.003.
